# diseaseGPS: auxiliary diagnostic system for genetic disorders based on genotype and phenotype

**DOI:** 10.1093/bioinformatics/btad517

**Published:** 2023-08-30

**Authors:** Daoyi Huang, Jianping Jiang, Tingting Zhao, Shengnan Wu, Pin Li, Yongfen Lyu, Jincai Feng, Mingyue Wei, Zhixing Zhu, Jianlei Gu, Yongyong Ren, Guangjun Yu, Hui Lu

**Affiliations:** State Key Laboratory of Microbial Metabolism, Joint International Research Laboratory of Metabolic & Developmental Sciences, Department of Bioinformatics and Biostatistics, School of Life Sciences and Biotechnology, Shanghai Jiao Tong University, Shanghai, China; SJTU-Yale Joint Center for Biostatistics and Data Science, National Center for Translational Medicine, Shanghai Jiao Tong University, Shanghai, China; State Key Laboratory of Microbial Metabolism, Joint International Research Laboratory of Metabolic & Developmental Sciences, Department of Bioinformatics and Biostatistics, School of Life Sciences and Biotechnology, Shanghai Jiao Tong University, Shanghai, China; SJTU-Yale Joint Center for Biostatistics and Data Science, National Center for Translational Medicine, Shanghai Jiao Tong University, Shanghai, China; Shanghai Children’s Hospital, School of Medicine, Shanghai Jiao Tong University, Shanghai, China; Shanghai Children’s Hospital, School of Medicine, Shanghai Jiao Tong University, Shanghai, China; Shanghai Engineering Research Center for Big Data in Pediatric Precision Medicine, Shanghai, China; Shanghai Children’s Hospital, School of Medicine, Shanghai Jiao Tong University, Shanghai, China; Shanghai Children’s Hospital, School of Medicine, Shanghai Jiao Tong University, Shanghai, China; Shanghai Children’s Hospital, School of Medicine, Shanghai Jiao Tong University, Shanghai, China; Shanghai Children’s Hospital, School of Medicine, Shanghai Jiao Tong University, Shanghai, China; Shanghai Children’s Hospital, School of Medicine, Shanghai Jiao Tong University, Shanghai, China; Shanghai Children’s Hospital, School of Medicine, Shanghai Jiao Tong University, Shanghai, China; Shanghai Engineering Research Center for Big Data in Pediatric Precision Medicine, Shanghai, China; State Key Laboratory of Microbial Metabolism, Joint International Research Laboratory of Metabolic & Developmental Sciences, Department of Bioinformatics and Biostatistics, School of Life Sciences and Biotechnology, Shanghai Jiao Tong University, Shanghai, China; SJTU-Yale Joint Center for Biostatistics and Data Science, National Center for Translational Medicine, Shanghai Jiao Tong University, Shanghai, China; State Key Laboratory of Microbial Metabolism, Joint International Research Laboratory of Metabolic & Developmental Sciences, Department of Bioinformatics and Biostatistics, School of Life Sciences and Biotechnology, Shanghai Jiao Tong University, Shanghai, China; SJTU-Yale Joint Center for Biostatistics and Data Science, National Center for Translational Medicine, Shanghai Jiao Tong University, Shanghai, China; Shanghai Children’s Hospital, School of Medicine, Shanghai Jiao Tong University, Shanghai, China; Shanghai Engineering Research Center for Big Data in Pediatric Precision Medicine, Shanghai, China; School of Medicine, The Chinese University of Hong Kong, Shenzhen, Guangdong, China; State Key Laboratory of Microbial Metabolism, Joint International Research Laboratory of Metabolic & Developmental Sciences, Department of Bioinformatics and Biostatistics, School of Life Sciences and Biotechnology, Shanghai Jiao Tong University, Shanghai, China; SJTU-Yale Joint Center for Biostatistics and Data Science, National Center for Translational Medicine, Shanghai Jiao Tong University, Shanghai, China; Shanghai Children’s Hospital, School of Medicine, Shanghai Jiao Tong University, Shanghai, China

## Abstract

**Summary:**

The next-generation sequencing brought opportunities for the diagnosis of genetic disorders due to its high-throughput capabilities. However, the majority of existing methods were limited to only sequencing candidate variants, and the process of linking these variants to a diagnosis of genetic disorders still required medical professionals to consult databases. Therefore, we introduce diseaseGPS, an integrated platform for the diagnosis of genetic disorders that combines both phenotype and genotype data for analysis. It offers not only a user-friendly GUI web application for those without a programming background but also scripts that can be executed in batch mode for bioinformatics professionals. The genetic and phenotypic data are integrated using the ACMG-Bayes method and a novel phenotypic similarity method, to prioritize the results of genetic disorders. diseaseGPS was evaluated on 6085 cases from Deciphering Developmental Disorders project and 187 cases from Shanghai Children’s hospital. The results demonstrated that diseaseGPS performed better than other commonly used methods.

**Availability and implementation:**

diseaseGPS is available to freely accessed at https://diseasegps.sjtu.edu.cn with source code at https://github.com/BioHuangDY/diseaseGPS.

## 1 Introduction

Genetic disorders are diseases caused by DNA variants on the genome, which afflicted ∼7.98% of the global population (Verma and Puri 2015). Despite this, more than 40% of the patients remained undiagnosed or misdiagnosed (Vandeborne *et al.* 2019). The next-generation sequencing has greatly improved the efficiency of genetic disorders diagnosis due to its high-throughput capabilities (Gong *et al.* 2018). Furthermore, the standardized and structured Human Phenotype Ontology (HPO) (Köhler *et al.* 2021) has made computer-based phenotype-driven disease diagnosis possible.

Several tools integrating phenotypes and genes have been developed for genetic disorder diagnosis. Among them, Phenomizer (Koehler *et al.* 2009) and Phenolyzer (Yang *et al.* 2015) computed a similarity score between a patient’s phenotype set and all the candidate diseases. Other tools such as eXtasy (Sifrim *et al.* 2013), Phen-Gen (Javed *et al.* 2014), Exomiser (Robinson *et al.* 2014), Xrare (Li *et al.* 2019), AMELIE (Birgmeier *et al.* 2020), and LIRICAL (Robinson *et al.* 2020) used existing *in silico* prediction algorithms to derive gene variant scores and combined these scores with phenotypic relevance scores for ranking disease-causing gene variants. However, when variant data were involved in these methods, the output was the ranking of candidate genes, and the final diagnosis of genetic disorders still required further confirmation.

Here, we present diseaseGPS, a platform that simultaneously analyzes both phenotypic and genetic data to conduct genetic disorder diagnosis. diseaseGPS provides a user-friendly and interactive graphical user interface (GUI) that is easy to use. It outperforms all the existing diagnosis tools on publicly available datasets from Deciphering Developmental Disorders (DDD) project and datasets from Shanghai Children’s hospital (SCH).

## 2 Method and implementation

diseaseGPS is a GUI web application based on the Browser/Server architecture. The published VCF-server (Jiang *et al.* 2019) is part of diseaseGPS for processing variant call format (VCF) files, whose overall framework is the same as diseaseGPS. The core functional modules of diseaseGPS are implemented in C and Python. The front-end of diseaseGPS is built with Sails.js based on the Node.js framework. And the back-end is built with PERL‐CGI. diseaseGPS accepts either or both of VCF files and HPO terms as input, since the two are analyzed independently. Users can upload VCF files and filter the variants based on annotative fields. They can also select HPO terms in the visual symptom tree or enter them manually.

**Figure 1.**
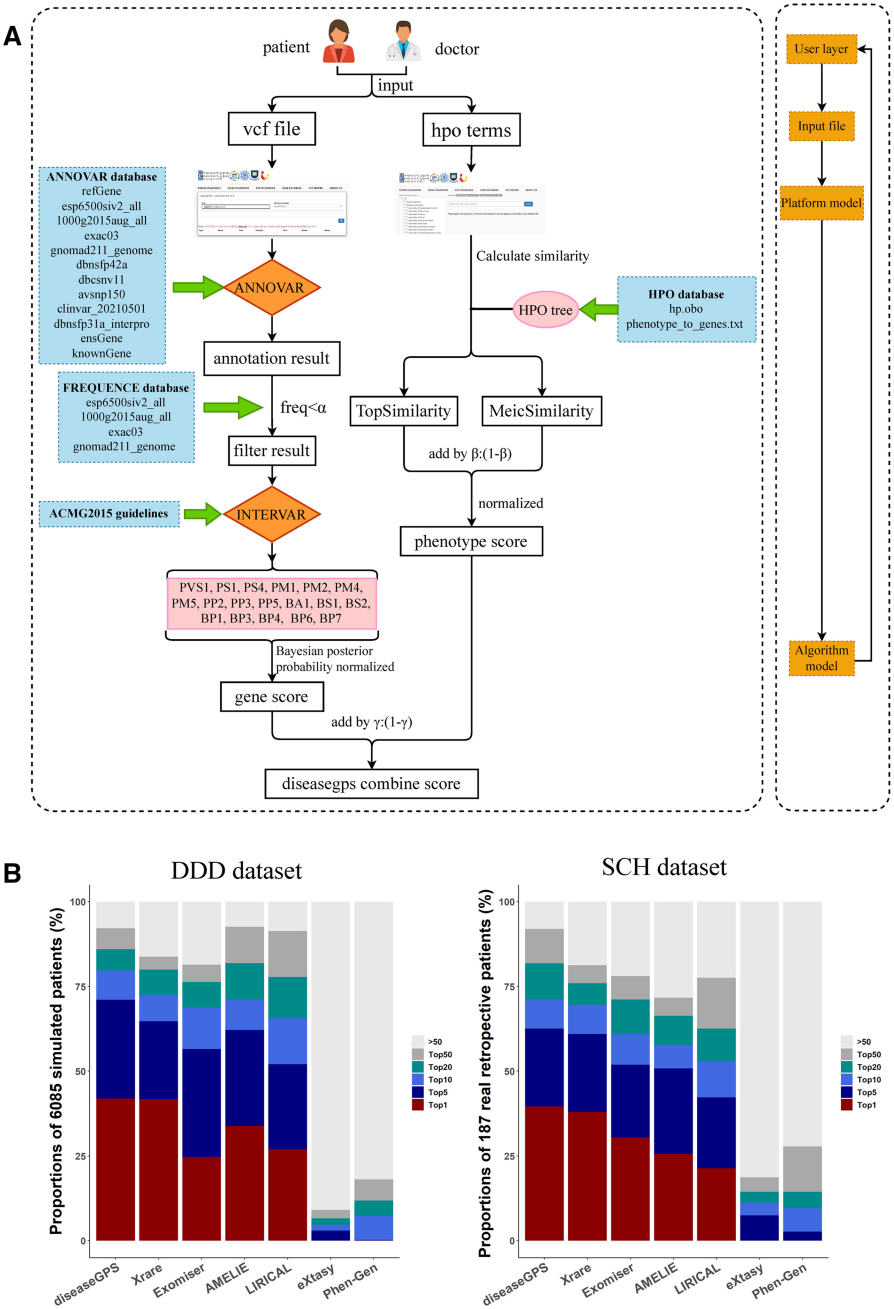
Workflow of diseaseGPS and results on DDD dataset and SCH dataset. (A) The workflow of diseaseGPS. After uploading the data, diseaseGPS will independently analyze VCF files and HPO terms for the prediction ranking results of genetic diseases. (B) Comparison of results of diseaseGPS with other tools on DDD dataset and SCH dataset. The abscissa shows the names of the various tools and the ordinate represents the proportion of cases with a causal outcome between TOP-1, TOP-5, TOP-10, TOP-20, TOP-50, and >50, respectively.

The workflow of diseaseGPS is shown in Fig. 1A. After uploading the data, diseaseGPS automatically analyzes the data to produce the predicted rankings of genetic disorders. For VCF files, diseaseGPS calls ANNOVAR (Wang *et al.* 2010) to generate annotations, including population allele frequency, conservation, pathogenicity, etc. Based on these annotations, users can set a screening threshold α for population allele frequency, which is set to 0.1 in this article. diseaseGPS also screens variants based on their inheritance mode. After the initial screening, diseaseGPS calls InterVar (Li and Wang 2017) to produce standard interpretations of sequence variants according to the ACMG-AMP guidelines (Richards *et al.* 2015). The evidence of automatic scoring contains one pathogenic very strong evidence (PVS1), two pathogenic strong evidence (PS1, PS4), four pathogenic moderate evidence (PM1, PM2, PM4, PM5), three pathogenic supporting evidence (PP2, PP3, PP5), one benign stand-alone evidence (BA1), two benign strong evidence (BS1, BS2), and five benign supporting evidence (BP1, BP3, BP4, BP6, BP7). diseaseGPS integrates the evidence of the ACMG-AMP guidelines using a Bayesian classification framework (Tavtigian *et al.* 2018) to calculate a unique gene score. The calculation formula for gene score can be found in the Supplementary File.

For inputted HPO terms, we use the topological similarity (TopSimilarity) and the semantic similarity (MeicSimilarity) to characterize phenotypic similarity between the patient’s phenotype set and the disease’s phenotype set. Based on the hp.obo file, we can build a tree structure diagram of HPO. The detailed calculation formulas for these two similarities can be found in the Supplementary File. The TopSimilarity and MeicSimilarity scores are summarized in a ratio of *β* to (1−*β*) and normalized to calculate the phenotype score. In this article, *β* is set to 0.9. The final diseaseGPS score is calculated by summing gene score and phenotype score in a ratio of *γ* to (1−*γ*). In this article, *γ* is set to 0.5.

## 3 Results

In this study, the phenotypic and genetic data of 6085 developmental disorder patients from the DDD project (Firth *et al.* 2009) was collected and used to evaluate the performance of diseaseGPS. The DDD dataset covered 1091 causative genes and 1743 genetic disorders listed in OMIM, providing a comprehensive representation of diseaseGPS’s universality. Since the whole exome sequencing data of DDD was not available, in-house exome sequencing data from 157 healthy individuals was used to regenerate the sequencing data as the background. In addition, the phenotypic and genetic data of 187 patients with confirmed genetic disorders from Children’s Hospital of Shanghai (SCH) was collected to demonstrate the effectiveness of diseaseGPS in real-world patients.

The parameters used to evaluate diseaseGPS are *α* = 0.1, *β* = 0.9 and *γ* = 0.5. To access the performance of diseaseGPS, it was compared to six other tools including Xrare, Exomiser, AMELIE, LIRICAL, Phen-Gen, and eXtasy. While diseaseGPS initially ranks causal diseases, it outputs the rankings of the causal genes through the background for intuitive comparison. The proportions of cases with a causal outcome at the levels of TOP-1, TOP-5, TOP-10, TOP-20, TOP-50, and >50 were calculated for each tool. The final results on DDD dataset and SCH dataset are shown in Fig. 1B. The results indicate that diseaseGPS outperforms other commonly used methods on both datasets. The TOP-1 ratio of diseaseGPS reached 41.89% on the DDD dataset and 39.57% on the SCH dataset. Wilcoxon signed-rank test revealed that diseaseGPS had significantly smaller rank numbers than Xrare (*P* < 2.2e−16 on the DDD dataset, *P* = 0.009587 on the SCH dataset). Another advantage of diseaseGPS is its user-friendly interface, offering both a convenient GUI web application for non-bioinformatics users and a command-line version for the convenience of bioinformatics personnel.

Since its launch on 27 March 2017, the web version of diseaseGPS has received 4963 access records from 56 countries and regions. The backend statistics show that diseaseGPS has received a total of 5575 valid retrievals.

## 4 Conclusions

We have developed diseaseGPS, an auxiliary diagnostic system that combines genotypic and phenotypic information to assist in the diagnosis of genetic disorders. diseaseGPS was evaluated on 6085 real cases from DDD dataset and 187 from SCH dataset with results showing that its superiority over most commonly used methods. diseaseGPS ranks all candidate genetic disorders by calculating phenotypic similarity and variant pathogenicity, effectively addressing the inaccuracies and noises present in both phenotypic data and genetic data. For more details on algorithms, results, and software information, please refer to the Supplementary File.

## Supplementary Material

btad517_Supplementary_Data

## Data Availability

The SCH data used in this article are not publicly available. One may contact the corresponding author (G.Y.) with reasonable requests. The DDD data underlying this article were provided by Deciphering Developmental Disorders project but restrictions apply to the availability of these data, and so are not publicly available.
