# Supplementary material for: diseaseGPS: auxiliary diagnostic system for genetic disorders based on genotype and phenotype
Source: Bioinformatics. 2023 Aug 30;39(9):btad517. doi: 10.1093/bioinformatics/btad517 (PMC10500091; doi:10.1093/bioinformatics/btad517)
Supplement: btad517_Supplementary_Data [file btad517_supplementary_data.docx]

**Supplemental data**

**diseaseGPS: auxiliary diagnostic system for genetic disorders based on genotype and phenotype**

Daoyi Huang^1†^, Jianping Jiang^1,3†^, Tingting Zhao^3†^, Shengnan Wu^2†^, Pin Li^2^, Yongfen Lyu^2^, Jincai Feng^2^, Mingyue Wei^2^, Zhixing Zhu^3^, Jianlei Gu^1^, Yongyong Ren^1^, Guangjun Yu^2*^ and Hui Lu^1,3*^

^1^ State Key Laboratory of Microbial metabolism, Joint International Research Laboratory of Metabolic & Developmental Sciences, Department of Bioinformatics and Biostatistics, SJTU-Yale Joint Center for Biostatistics and Data Science, School of Life Sciences and Biotechnology, Shanghai Jiao Tong University, Shanghai, China

^2^ Shanghai Children’s Hospital, School of medicine, Shanghai Jiao Tong University, Shanghai, China

^3^ Shanghai Engineering Research Center for Big Data in Pediatric Precision Medicine, Shanghai Children’s Hospital, School of medicine, Shanghai Jiao Tong University, Shanghai, China

^†^ These authors contributed equally.

^*^ corresponding author.

**Correspondent:**

Hui Lu, Department of Bioinformatics and Biostatistics, SJTU-Yale Joint Center for Biostatistics, School of Life Sciences and Biotechnology, Shanghai Jiao Tong University, 800 Dongchuan Road, Minhang District, Shanghai, China; Email: [huilu@sjtu.edu.cn](mailto:huilu@sjtu.edu.cn)

Guangjun Yu, Shanghai Children's Hospital, School of medicine, Shanghai Jiao Tong University, 24 West Beijing Road, Lane 1400, Shanghai, China; Email: [gjyu@shchildren.com.cn](mailto:gjyu@shchildren.com.cn)

**• Supplementary Methods**

**•** **Supplementary Figures (S1-S22)**

**• Supplementary Discussion**

**• Supplementary References**

**Supplementary Methods**

**The manual of GUI web application for diseaseGPS**

The graphical user interface (GUI) web application of diseaseGPS offers four main functions: phenotype-driven genetic disorder diagnosis (PHENO-DIAGNOSIS), genotype-driven genetic disorder diagnosis (GENO-DIAGNOSIS), synthesized phenotype- and gene-driven genetic disorder diagnosis (SYN-DIAGNOSIS), and case database management (CASE DATABASE). Users can select the required function from the home page according to the type of data available.

**Source and preprocessing of test data**

A large number of methods combine simulated data and real data to test algorithms. Using a relatively fair approach, Yuan et al. (Yuan, et al., 2022) benchmarked ten existing genetic sequencing methods, including 305 cases from Deciphering Developmental Disorders (DDD) (Fitzgerald, et al., 2015) project and 209 in-hospital cases. Based on their benchmark results, we chose to reproduce the six methods that performed well to compare with diseaseGPS. The test dataset was divided into two parts: the simulated dataset and the real dataset.

The simulated dataset was downloaded from the Deciphering Developmental Disorders (DDD) study in the European Genome-Phenome Archive (EGA) (Lappalainen, et al., 2015) study EGAS00001000775. The downloaded simulated dataset contains 8558 cases with Single Nucleotide Variation, as well as VCF files, causal genes, and phenotypes in Human Phenotype Ontology (HPO) (Robinson, et al., 2008) terms. 6085 cases were screened in Online Mendelian Inheritance in Man (OMIM) (Hamosh, et al., 2005) with a definitive diagnosis of genetic disorder based on causal genes and their inheritance modes. The variant loci in each case were randomly assigned to one of 157 healthy VCFs as the background genome. The VCF files of 157 healthy people were provided by Shanghai Children's Hospital and were independent and unrelated to the DDD study. We call this simulated dataset the DDD dataset.

The real data was provided by Shanghai Children's Hospital (SCH), which included data of 187 patients with genetic disorders. Unlike the simulated data, the real data contains complete VCF files, disease-causing genes, and HPO terms. We call this real heterozygous dataset the SCH dataset.

**Variants screening based on inheritance mode**

One characteristic of genetic disorders is that they have different inheritance modes. Since not all variant sites are pathogenic, diseaseGPS screens for variant sites based on inheritance mode. diseaseGPS obtained the inheritance modes for all diseases from the OMIM database (Hamosh, et al., 2005). diseaseGPS can identify both recessive and dominant inheritance modes, as well as the compound inheritance pattern. To determine whether a variant is disease-causing, all variants are first screened for compatibility with recessive or dominant inheritance. For unmatched variants, a second step is taken to assess compound heterozygosity. A variant in a gene is considered to meet the conditions of compound heterozygosity if there are two or more non-intronic or non-synonymous heterozygous variants in that gene.

**The phenotypic similarity on the human phenotype ontology hierarchy**

Existing phenotype similarity algorithms are based on the hierarchical structure of the phenotype tree, which is derived from set-based similarity algorithms (Blanchard, et al., 2008). In diseaseGPS, we use topological similarity ($TopSimilarity$) and semantic similarity ($MeicSimilarity$) to characterize phenotypic similarity. The phenotype set entered by users is marked as $v_{1}$, and the annotated phenotype set of any OMIM disease is marked as $v_{2}$. Based on the hp.obo file, a tree structure diagram of HPO can be constructed. $TopSimilarity$ can be calculated according to equation (1):

$$TopSimilarity\left( v_{1},v_{2} \right)=\frac{Top(v_{1}\cap v_{2})}{Top(v_{1})} (1)$$

Where $Top(v)$ refers to the number of ancestors of set $v$. $MeicSimilarity$ can be calculated according to equation (2):

$$MeicSimilarity\left( v_{1},v_{2} \right)=\max_{p\in v_{1}\cap v_{2}} \ln\left( \frac{N}{n_{p}} \right)\times ancestors(p) (2)$$

Where $N$ is the total number of OMIM diseases, $n_{p}$ is the number of OMIM diseases annotated by phenotype$p$, $ancestors(p)$ is the number of ancestor nodes of phenotype $p$. The $TopSimilarity$ and $MeicSimilarity$ scores are summarized in a ratio of β to (1-β) and normalized to calculate the phenotype score. In this paper, β is set to 0.9.

The phenotype-driven diseaseGPS score provides a p-value obtained through permutation test to indicate its significance. Given a user-provided set of phenotypes $v_{u}$, with a total of $N_{u}$ phenotypes, and a specific genetic disease $\mathcal{D}$, $S\left( v_{u}\mathcal{,D} \right)$ represents the diseaseGPS score computed based on phenotype similarity. The calculation steps are as follows:

1. Randomly select HPO terms from all HPO terms excluding the root term "ALL" to form a random phenotype set $v_{r1}$, with a quantity of $N_{u}$.
2. Calculate the phenotype-driven diseaseGPS score between the random phenotype set $v_{r1}$ and genetic disease $\mathcal{D}$, denoted as $S\left( v_{r1}\mathcal{,D} \right)$.
3. Repeat step① and step② 1000 times to obtain a random score set $( S\left( v_{r1}\mathcal{,D} \right), S\left( v_{r2}\mathcal{,D} \right), \ldots,S\left( v_{r1000}\mathcal{,D} \right) )$.
4. Sort this random score set in ascending order to construct a sampling distribution, denoted as $(S_{1},S_{2},\ldots,S_{1000})$.
5. Check if $S\left( v_{u}\mathcal{,D} \right)$ falls within the confidence interval of the distribution $(S_{1},S_{2},\ldots,S_{1000})$. Assuming that there are $N_{p}$ values greater than $S\left( v_{u}\mathcal{,D} \right)$ in the random score set, the final p-value of $S\left( v_{u}\mathcal{,D} \right)$ is $N_{p}/1000$.

In actual clinical scenarios, doctors usually observe only superficial phenotypes, which are also known as imprecise phenotypes. Due to the characteristics of information content, the information content of child nodes is always higher than that of parent nodes, resulting in excessive penalties for imprecise phenotypes. For example, the Human Phenotype Ontology divides the phenotype "HP: 0001249, Intellectual disability" into six detailed categories: "HP: 0001256, Mild intellectual disability", "HP: 0002342, Moderate intellectual disability", "HP: 0010864, Severe intellectual disability", "HP:0002187, Profound intellectual disability", "HP:0006887, Progressive intellectual disability" and "HP:0006889, Borderline intellectual disability". In medical records, doctors often record "intellectual disability" without specifying its severity or progression. However, the penalty mechanism of the previous similarity algorithm would result in an overly high penalty for this common occurrence. We used topological similarity to measure the similarity of two sets of phenotypes, as it penalizes all nodes equally. In order to make up for the lack of information in imprecise phenotypes, we introduced MeicSimilarity. MeicSimilarity refers to the phenotype node with the highest amount of information content in the intersection of the patient phenotype set and the disease-annotated phenotype set. Typically, this phenotype node represents the most specific phenotype, which is useful for physicians in making an initial diagnosis and subsequent differential diagnoses.

**The genetic pathogenicity score based on Bayesian classification framework**

In 2015, the American College of Medical Genetics and Genomics (ACMG) and the Association for Molecular Pathology (AMP) published the standards and guidelines for the interpretation of sequence variants based on 28 criteria for evidence evaluation (Richards, et al., 2015). The ACMG-AMP guidelines provided standardized criteria for determining gene pathogenicity, unifying the diverse approaches used worldwide. diseaseGPS integrates the evidence of the ACMG-AMP guidelines using a Bayesian classification framework (Tavtigian, et al., 2018) to calculate a unique gene score. The odds of pathogenicity are calculated by equation (3):

$$OddsPath={O_{PVSt}}^{(\frac{N_{PSu}}{8}+\frac{N_{PM}}{4}+\frac{N_{PSt}}{2}+\frac{N_{PVSt}}{1}-\frac{N_{BSu}}{8}-\frac{N_{BSt}}{1})} (3)$$

Among them, $N_{PSu},N_{PM},N_{PSt},N_{PVSt},N_{BSu}$ and $N_{BSt}$ are the number of PP, PM, PS, PVS, BP and BS evidences, respectively. $O_{PVSt}$ is the pathogenic strength of strong evidence of pathogenicity, which defaults to 350. $OddsPath$ is the conditional probability of pathogenicity for a gene variant site. The posterior probability of pathogenic is expressible as a specific example of equation (4):

$$Post_{P}=\frac{OddsPath\times Prior_{P}}{\left( \left( OddsPath-1 \right)\times Prior_{P}+1 \right)} (4)$$

${Prior}_{P}$ is the prior probability of the pathogenicity of the gene variant site, which defaults to 0.1. ${Post}_{P}$ is the posterior probability of the pathogenicity of the gene variant site and is used as the gene score.

**Integration of phenotypic similarity and genetic pathogenicity score**

The final results obtained by diseaseGPS are the predicted scores and rankings of candidate genetic disorders. For a specific genetic disorder $\mathcal{D}$, its phenotype score $S_{phenotype}\left( \mathcal{D} \right)$ and gene score $S_{gene}\left( \mathcal{D} \right)$ are calculated by equation (5) and (6), respectively:

$$S_{phenotype}\left( \mathcal{D} \right)=\beta\times TopSimilarity\left( v_{u},v_{\mathcal{D}} \right)+\left( 1-\beta\right)\times MeicSimilarity\left( v_{u},v_{\mathcal{D}} \right) (5)$$

Where $v_{u}$ refers to the set of phenotypes entered by the user, and $v_{\mathcal{D}}$ refers to the annotated phenotype set of genetic disorder $\mathcal{D}$.

$$S_{gene}\left( \mathcal{D} \right)=\max_{\mathcal{w\in W(D)}} Post_{P}\mathcal{(w)} (6)$$

Where $\mathcal{W(D)}$ refers to the set of all variants associated with the gene linked to genetic disorder $\mathcal{D}$.

For a specific genetic disorder $\mathcal{D}$, its final diseaseGPS prediction score is obtained by equation (7):

$$S_{diseaseGPS}\left( \mathcal{D} \right)=\gamma\times S_{phenotype}\left( \mathcal{D} \right)+\left( 1-\gamma\right)\times S_{gene}\left( \mathcal{D} \right) (7)$$

However, the final results of other methods are the ranking of candidate genes. In order to ensure fairness and accuracy of comparison, we use the ranking of genes output in the background for result comparison. For a specific gene $\mathcal{G}$, if there are still potentially pathogenic variants on it after filtering, and $\mathcal{D}_{\mathcal{G}}$ is a genetic disorder associated with gene $\mathcal{G}$, then the gene $\mathcal{G}$ is assigned the phenotype score of the genetic disorder $\mathcal{D}_{\mathcal{G}}$, denoted as $S_{phenotype}(\mathcal{G}_{\mathcal{D}})$, which is expressed as equation (8):

$$S_{phenotype}\left( \mathcal{G}_{\mathcal{D}} \right)=\left\{ \begin{aligned} S_{phenotype}\left( \mathcal{D}_{\mathcal{G}} \right) if pathogenic variant on gene \mathcal{G} \\ 0 otherwise \end{aligned} (8) \right.$$

For a specific gene $\mathcal{G}$, its phenotype score $S_{phenotype}\left( \mathcal{G} \right)$ and gene score $S_{gene}\left( \mathcal{G} \right)$ are calculated by equation (9) and (10), respectively:

$$S_{phenotype}\left( \mathcal{G} \right)= \max_{D\mathcal{'\in M(}\mathcal{G})}S_{phenotype}\left( {D'}_{\mathcal{G}} \right) (9)$$

$$S_{gene}\left( \mathcal{G} \right)=\max_{\mathcal{w\in W(}\mathcal{G})} Post_{P}\mathcal{(w)} (10)$$

Where $\mathcal{M(}\mathcal{G})$ refers to the set of genetic disorders associated with the gene $\mathcal{G}$. And $\mathcal{W(}\mathcal{G})$ refers to the set of all variants associated with the gene $\mathcal{G}$.

For a specific gene $\mathcal{G}$, its final diseaseGPS prediction score is obtained by equation (11):

$$S_{diseaseGPS}\left( \mathcal{G} \right)=\gamma\times S_{phenotype}\left( \mathcal{G} \right)+\left( 1-\gamma\right)\times S_{gene}\left( \mathcal{G} \right) (11)$$

It should be noted that the predicted scores and rankings for candidate genes are only used for comparison with other algorithms, and the actual output on the web server is the predicted scores and rankings for candidate genetic disorders.

**The phenotype-driven diagnostic workflow of diseaseGPS**

The web interface for phenotypic diagnosis of diseaseGPS is shown as Figure S2. Users have the option to select the patient's phenotype in the phenotype tree or manually enter the phenotype in the input box. Users can input HPO terms or textual descriptions for phenotypes. If a user provides an incomplete textual description, corresponding matching suggestions will be generated to assist the user in selecting the corresponding HPO term. It is worth noting that the root node of the phenotype tree is ALL. The closer the phenotype is to the leaf node, the more accurate it may be, leading to a potentially higher prediction accuracy. Users can view the detailed information of the phenotypes entered in the phenotype information sheet. After entering all the phenotypes of the patient, Users can click the "Search" button to obtain the diagnosis results of phenotype-driven genetic disorders.

The results of phenotype-driven genetic disorder diagnosis are shown in Figure S3. The results include the name of the genetic disorder, the predicted diseaseGPS score and its p-value as well as the correlation between the inputted phenotype and the genetic disorder. The results also include a description of the genetic disorder, the causative gene associated with the genetic disorder, its mode of inheritance, age of death, prevalence, indel, clinical modifier, onset, and the ID of the genetic disorder in the OMIM database.

Users can click on each entry in Figure S3 to obtain more detailed results, including detailed information on genetic disorders, a diagram of individual phenotype, detailed information on causative genes, and a complete diagram of all input phenotypes, as shown in Figure S4, S5, S6 and S7, respectively.

**The genotype-driven diagnostic workflow of diseaseGPS**

The web interface for genotypic diagnosis of diseaseGPS is shown as Figure S8. Users can upload VCF files to the box and select the genome version. After uploading VCF files, users can click the "OK" button to obtain the diagnosis results of genotype-driven genetic disorders. Users can also click the "Analyze" box to obtain the annotation analysis result of the VCF file, as shown in Figure S9. Additionally, users can click the "Diagnose" box to obtain the disease diagnosis prediction results of the VCF file, as shown in Figure S13. In the analysis results of the VCF file in Figure S9, users can customize the columns, filter the entries and perform settings through the "Display", "Filter", and "Setting" buttons, as shown in Figure S10, S11, and S12. In the diagnostic results of Figure S13, the gene-driven diagnostic results increase ACMG-based causative genes and their pathogenic evidence compared to the phenotype-driven diagnostic results.

**The phenotype and genotype co-driven diagnostic workflow of diseaseGPS**

The comprehensive diagnostic interface driven by phenotype and genotype is shown in Figure S14. Users can upload a VCF file and enter a patient's phenotype collection simultaneously. After the user's operation is completed, a diagnosis result that integrates the phenotype data and the genotype data can be obtained, as shown in Figure S15. The information in the comprehensive diagnostic results contains all the details shown in Figure S3 and Figure 13.

**Case database management**

After logging into their account, users are able to create their own case database. The case creation interface is shown in Figure S16. Users can enter patient-related information such as gender, age, description, phenotype, diagnosed disease, and more. Users can choose to keep the case private, so that other users cannot view the detailed information of the case. Alternatively, users can make the case public, so that all users can view it. If users want to obtain relevant private case information, they can request access from the case owner. The construction of the case database enables users to manage their own patients in a more organized manner within diseaseGPS, while also providing a way for sharing information among related patients.

**The version and source of the tools being tested**

| Tool | Source | Version |
| --- | --- | --- |
| Xrare (Li, et al., 2019) | https://web.stanford.edu/~xm24/Xrare/ | pub:2015 |
| Exomiser (Robinson, et al., 2014) | https://github.com/exomiser/Exomiser | 12.1.0 |
| AMELIE (Birgmeier, et al., 2020) | https://amelie.stanford.edu/ | 3.1.0. |
| LIRICAL (Robinson, et al., 2020) | https://github.com/TheJacksonLaboratory/LIRICAL | v2.0.0-RC1 |
| eXtasy (Sifrim, et al., 2013) | https://github.com/asifrim/eXtasy/ | 0.1 |
| Phen-Gen (Javed, et al., 2014) | https://github.com/pkuerten/phen-gen/ | initial version |

**The allele frequency cutoff used by the tools**

| Tool | Allele frequency cutoff | Database |
| --- | --- | --- |
| diseaseGPS | 0.1 | 1000 Genomes Project, Exome Server Project (ESP), Exome Aggregation Consortium (ExAC) and Genome Aggregation Database (gnomAD) |
| Xrare (Li, et al., 2019) | 0.05 | Exome Aggregation Consortium (ExAC) |
| Exomiser (Robinson, et al., 2014) | 0.01 | 1000 Genomes Project, Database of Single Nucleotide Polymorphisms (dbSNP), Exome Server Project (ESP) and Exome Aggregation Consortium (ExAC) |
| AMELIE (Birgmeier, et al., 2020) | 0.005 | Genome Aggregation Database (gnomAD) |
| LIRICAL (Robinson, et al., 2020) | 0.01 | 1000 Genomes Project, Database of Single Nucleotide Polymorphisms (dbSNP), Exome Server Project (ESP) and Exome Aggregation Consortium (ExAC) |
| eXtasy (Sifrim, et al., 2013) | 0.01 | 1000 Genomes Project |
| Phen-Gen (Javed, et al., 2014) | 0.01 | 1000 Genomes Project, National Heart, Lung, and Blood Institute Exome Sequencing Project (NHLBI ESP) and Database of Single Nucleotide Polymorphisms (dbSNP) |

The allele frequency cutoff in the table represents the default parameter setting for all tools. In addition to setting allele frequency cutoffs for variant filtering, some tools also employ additional filtering criteria. For the sake of fairness in comparison, all tools were tested with default parameters and selected to display all results.

**Supplementary Figures (S1-S22)**

**
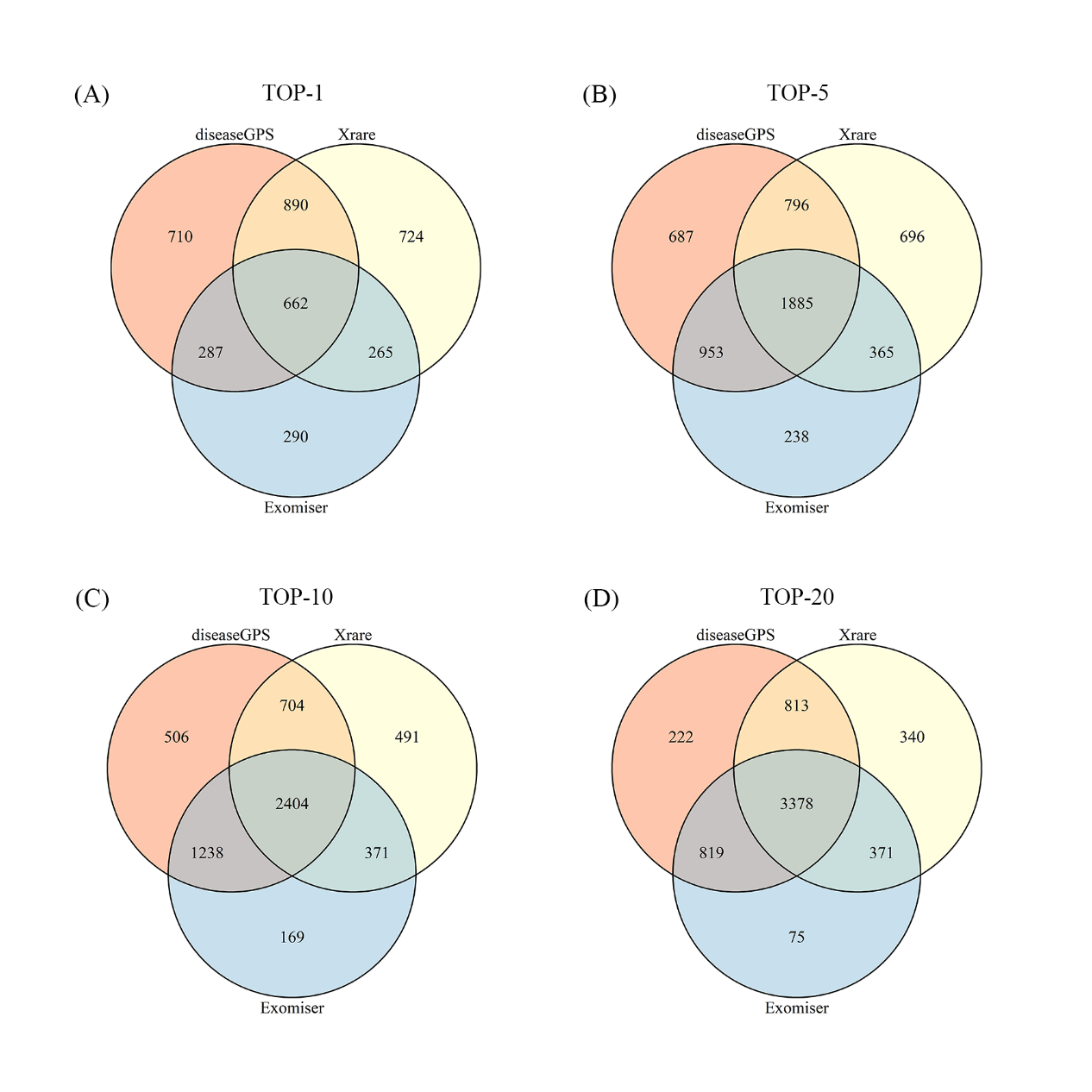
**

**Figure S1.** Venn diagram showing shared and unique TOP distribution of prediction results between diseaseGPS, Xrare(Li, et al., 2019) and Exomiser(Robinson, et al., 2014) on the DDD dataset. **(A) (B) (C) (D)** are Venn diagrams of the TOP-1, TOP-5, TOP-10, TOP-20 distribution of diseaseGPS, Xrare and Exomiser on the DDD dataset, respectively. The number of shared results among the three methods is lower in TOP-1, and the gradually increases from TOP-5 to TOP-10, and then to TOP-20.

**
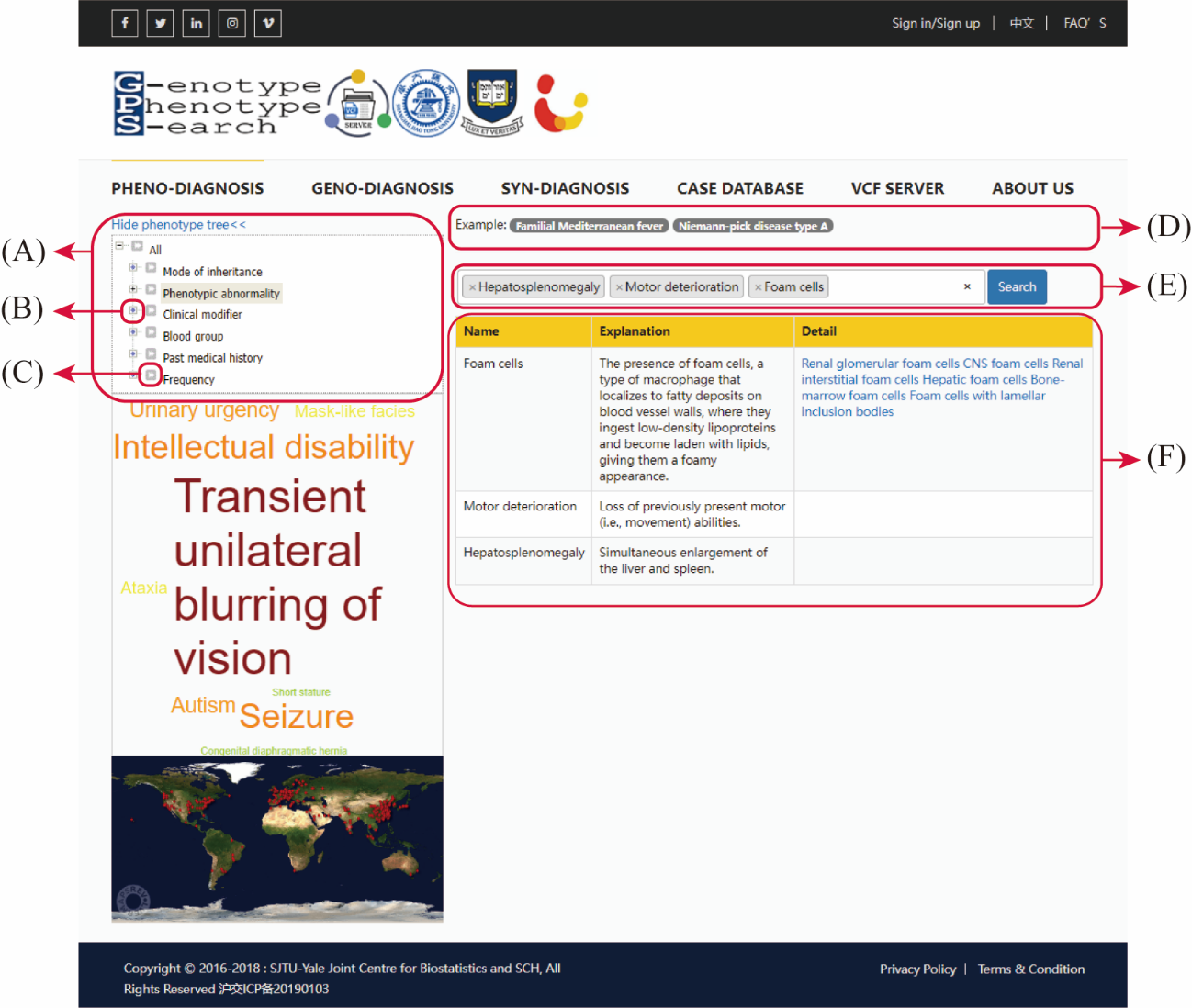
**

**Figure S2.** The web interface for phenotypic diagnosis of diseaseGPS. **(A)** The phenotype tree. **(B)** The expand button. Users can click on the "•" icon to the left of phenotype term to obtain more detailed phenotypes. **(C)** The add button. Users can click on the white double arrow with gray background to add phenotype term to the phenotype input box. **(D)** The example genetic disorder. Users can click on "Familial Mediterranean fever" or "Niemann-pick disease type A" in the examples, and the corresponding HPO terms associated with these two genetic diseases will be automatically filled in the phenotype input box. **(E)** The phenotype input box. Users can search by manually entering phenotypes in the phenotype input box. **(F)** The phenotype information sheet. The phenotypic information contains basic explanations and details of phenotypes. After entering all the phenotypes of the patient, users can click the "Search" button to obtain the diagnosis results of phenotype-driven genetic disorders.


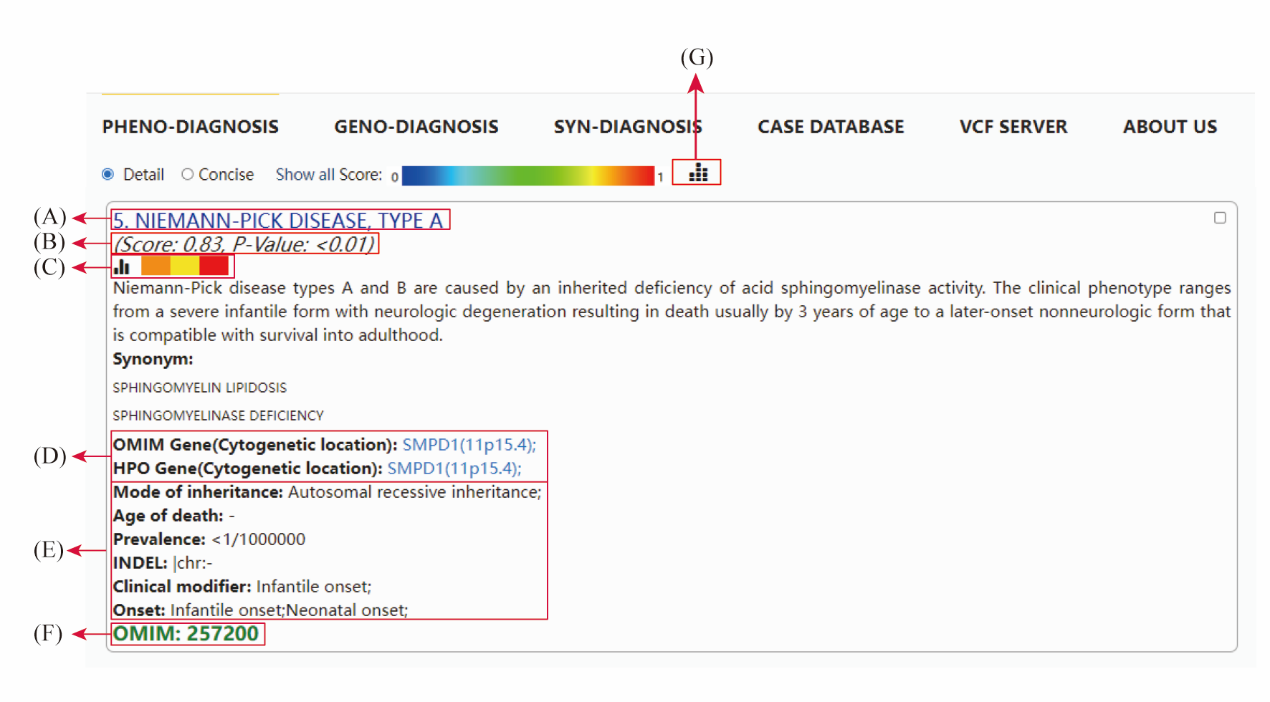


**Figure S3.** An example of phenotype diagnosis of diseaseGPS are displayed, whose input phenotypes are Hepatosplenomegaly (HP:0001433), Motor deterioration (HP:0002333) and Foam cells (HP:0003651). **(A)** The name of a genetic disorder and its ranking among all genetic disorders. Users can click on this item to obtain more detailed information about the genetic disorder, as shown in Figure S4. **(B)** The diseaseGPS score of the genetic disorder and its p-value. **(C)** The correlation between the inputted phenotype and the genetic disorder. As the color changes from blue to red, its correlation increases gradually. Users can click on one of the color blocks to obtain the phenotype relation diagram of the phenotype in the entire phenotype tree, as shown in Figure S5. **(D)** The causative gene associated with the genetic disorder. Users can click on this item to obtain more detailed information about the causative gene, as shown in Figure S6. **(E)** More information about the genetic disorder, including mode of inheritance, age of death, prevalence, indel, clinical modifier and onset. **(F)** The ID of the genetic disorder in the OMIM database. Users can click on the item to hyperlink to the OMIM database for more information. **(G)** After selecting a genetic disorder item, users can see this icon. Users can click on the item to access a network diagram of the selected genetic disorder and inputted phenotype set, as shown in Figure S7.


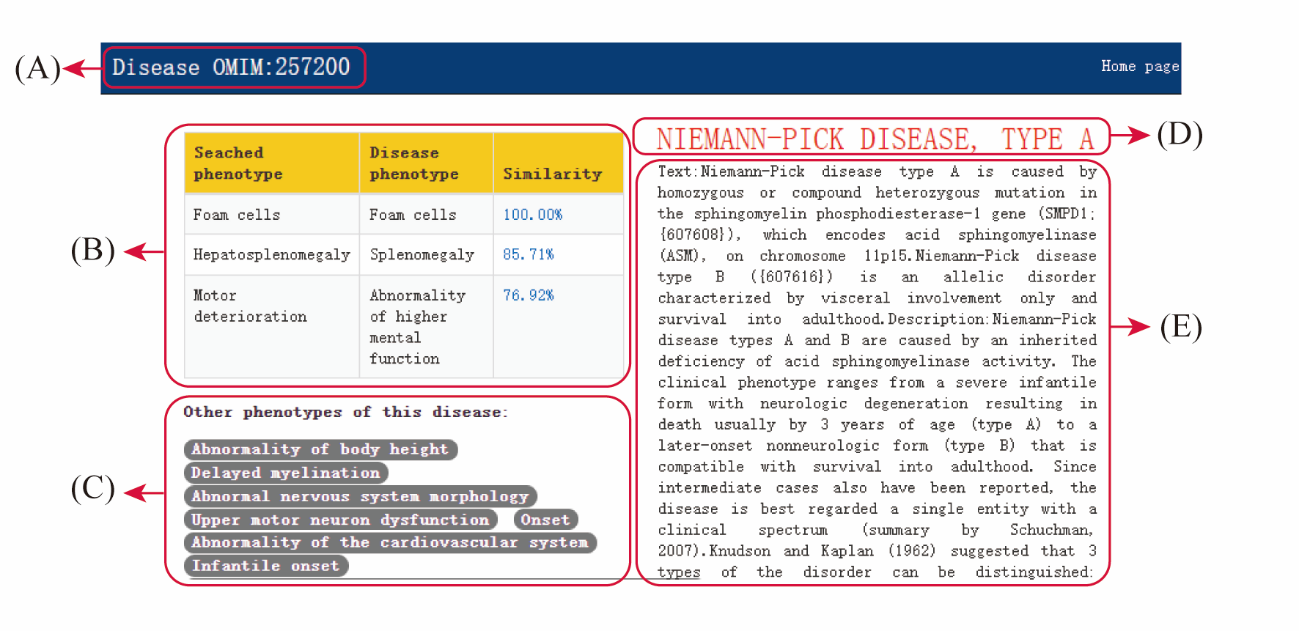


**Figure S4.** An example of genetic disorder details for phenotype diagnosis of diseaseGPS. **(A)** The ID of the genetic disorder in the OMIM database. **(B)** The most similar part of the searched phenotype to the annotated phenotype of the genetic disorder and the similarity score. **(C)** Other unmatched phenotypes in this genetic disorder. Users can revisit patients for matching phenotypes and click to add. **(D)** The name of the genetic disorder. **(E)** The textual description of the genetic disorder.

**
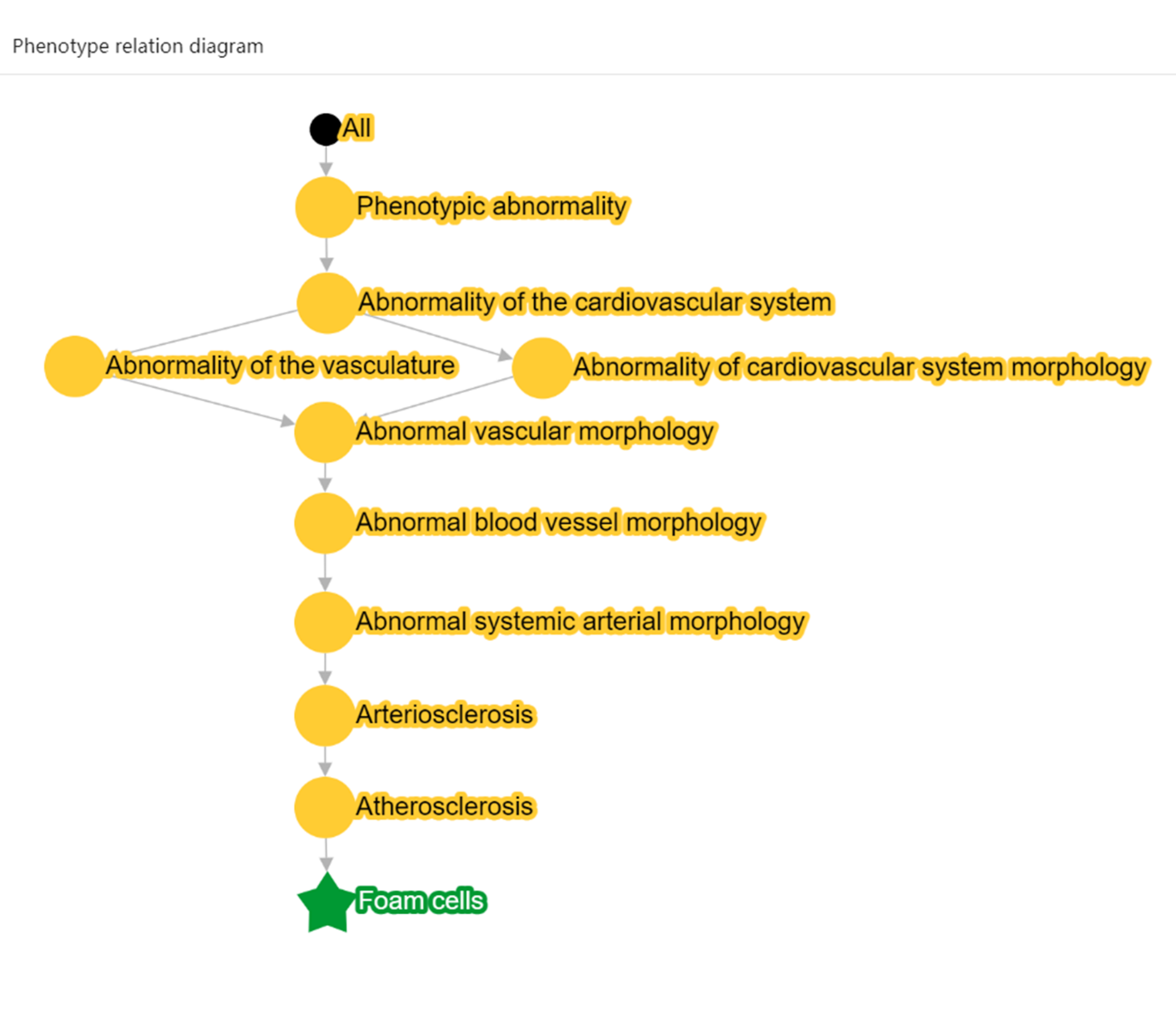
**

**Figure S5.** An example of phenotype relation diagram for phenotype diagnosis of diseaseGPS. The black circle is the root node of all phenotype nodes. The green star is the selected phenotype node. The yellow circles are the ancestor nodes of the selected phenotype node. The arrows represent the "is_a" relationships between the phenotype nodes.

**
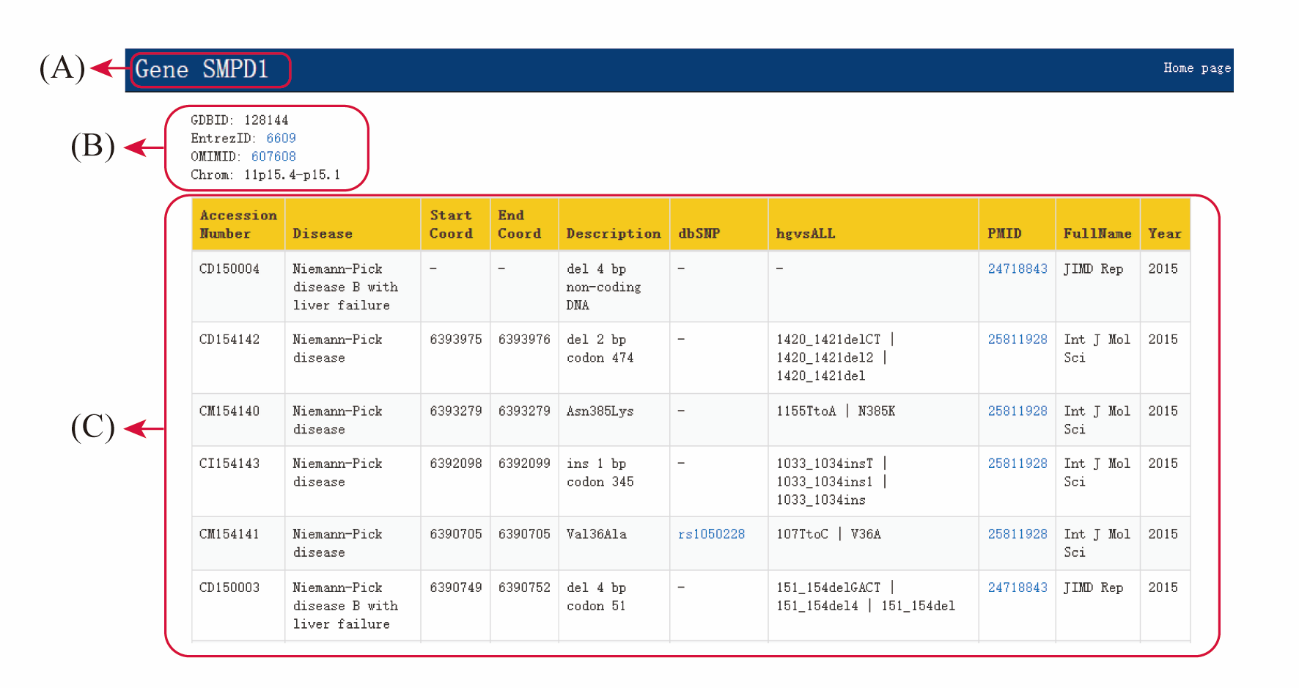
**

**Figure S6.** An example of causative gene details for phenotype diagnosis of diseaseGPS. **(A)** The name of the causative gene. **(B)** The brief information on the causative gene, including GDB ID, Entrez ID, OMIM ID and Chromosome. **(C)** The variant sites present on this gene and their details, including accession number, related disease, start coordinate, end coordinate, description, dbSNP, hgvsALL, PMID, full name and year.


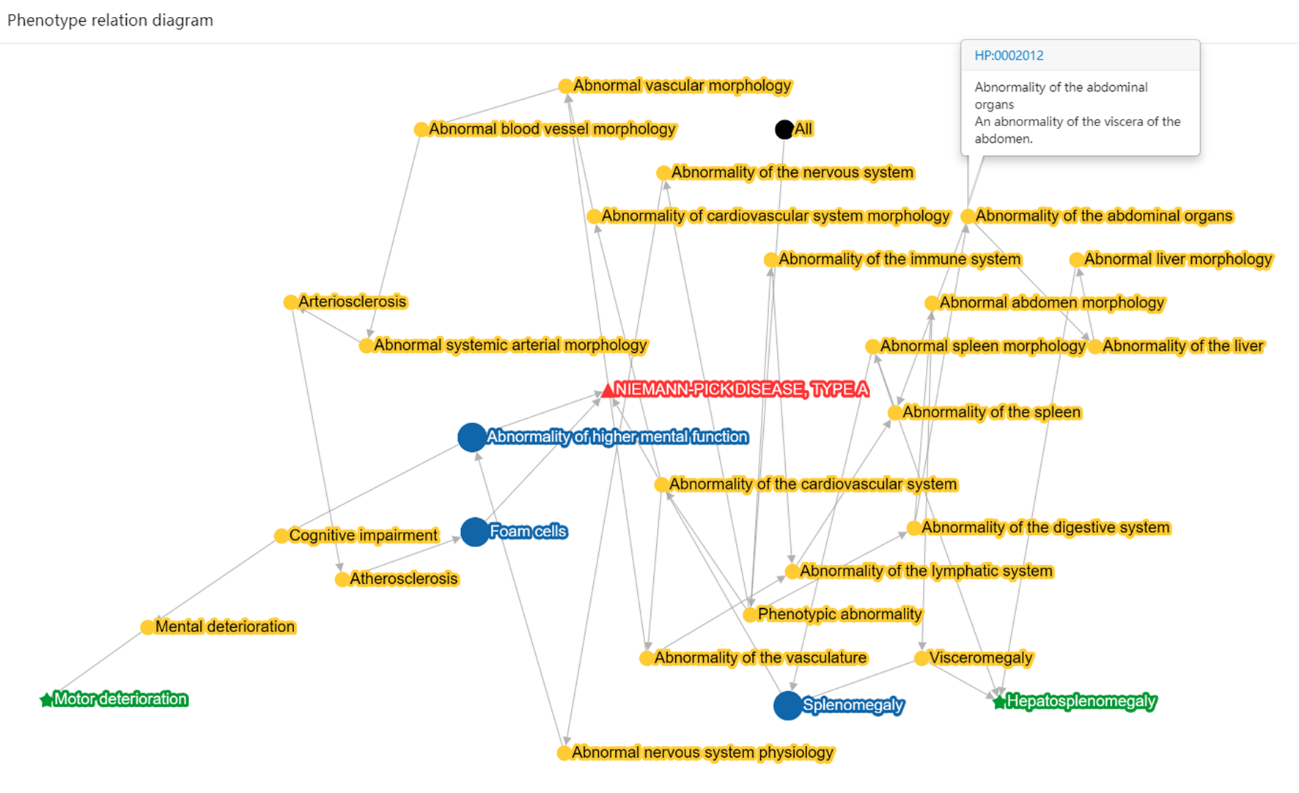


**Figure S7.** An example of complete phenotype relation diagram for phenotype diagnosis of diseaseGPS. The black circle is the root node of all phenotype nodes. The green stars are the inputted phenotype nodes. The yellow circles are the ancestor nodes of the inputted phenotype nodes. The red triangle is the selected genetic disorder. The blue circles in the annotated phenotype set for the genetic disorder represent the phenotypes that are most similar to the inputted phenotype set. The arrows represent the "is_a" relationships between the phenotype nodes or annotated relationships between disease and phenotype.

**
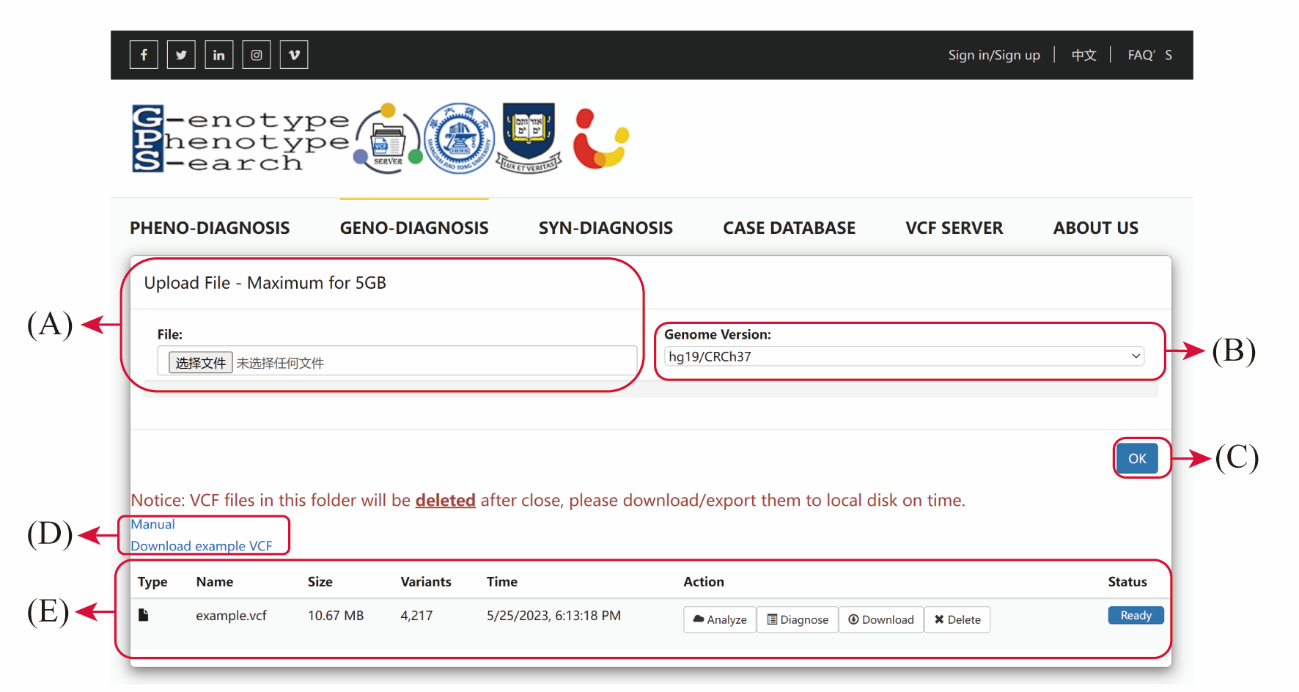
**

**Figure S8.** The web interface for genotypic diagnosis of diseaseGPS. **(A)** The box for uploading VCF files. diseaseGPS accepts VCF files or vcf.gz files up to 5GB in size. **(B)** The genome version selection box. Users can choose hg19/CRCh37 or hg38/CRCh38 according to the annotated genome of VCF file. **(C)** After users click the "OK" box, diseaseGPS starts to analyze the VCF file. **(D)** The example VCF file and user manual. Users can click to download the example file and follow the manual for instructions on how to use it. **(E)** The summary list of information for VCF files, including file type, file name, file size, the number of variants in the file, upload time, actions users can perform and analysis status. Users can click the "Analyze" box to obtain the annotation analysis results of the VCF file, as shown in Figure S9. Users can click the "Diagnose" box to obtain the disease diagnosis prediction results of the VCF file, as shown in Figure S13.


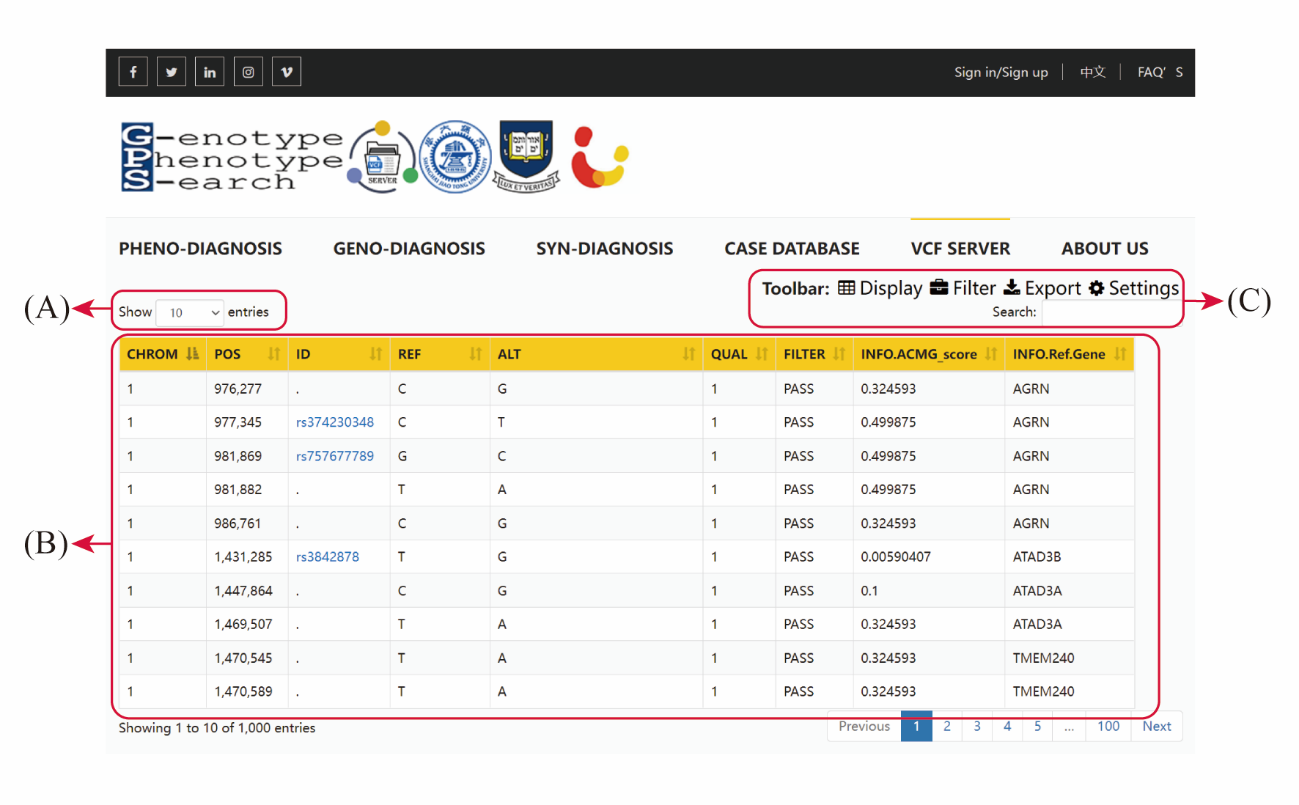


**Figure S9.** An example of analysis result for genotypic diagnosis of diseaseGPS. **(A)** The selection box showing the number of entries. **(B)** Users can customize the columns through "Display" in the toolbar, as shown in Figure S10. Users can filter the entries through "Filter" in the toolbar, as shown in Figure S11. Users can perform settings through "Setting" in the toolbar, as shown in Figure S12. In addition, users can also enter information in the "Search" box to conduct a query. **(C)** The list of analysis results for genotypic diagnosis of diseaseGPS. Users can modify the displayed results through the toolbar, and sort or reverse sort columns by clicking its name.


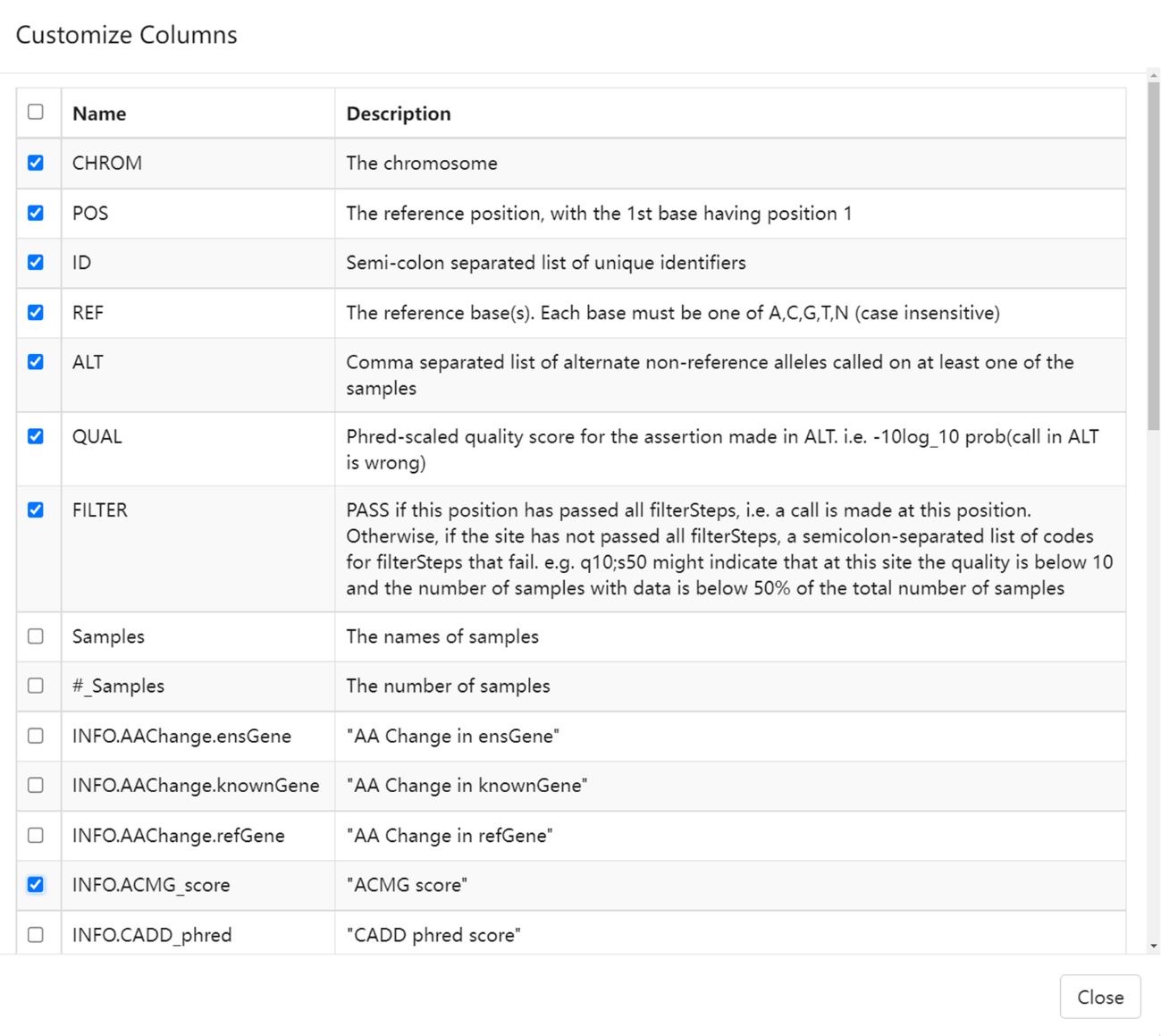


**Figure S10.** Customize columns to display results for genotypic diagnosis of diseaseGPS. The initial column contains basic information from the VCF file, and users can add additional columns with information that they are interested in.

**
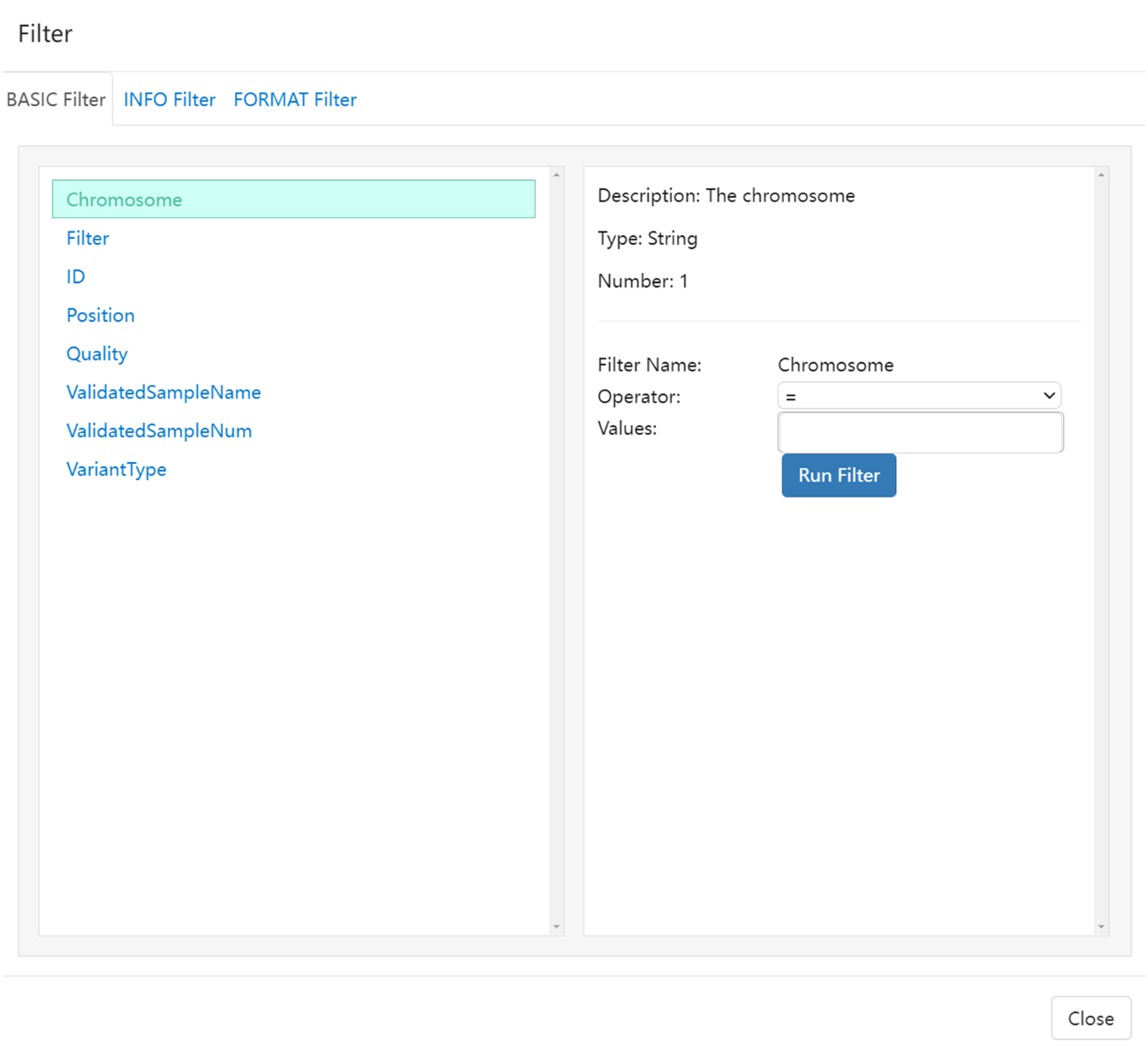
**

**Figure S11.** Filter entries to display results for genotypic diagnosis of diseaseGPS. Users can filter numerical values in columns or text in string columns. Filtering is divided into three categories: basic information from the VCF file, annotation information, and format information of zygosity.


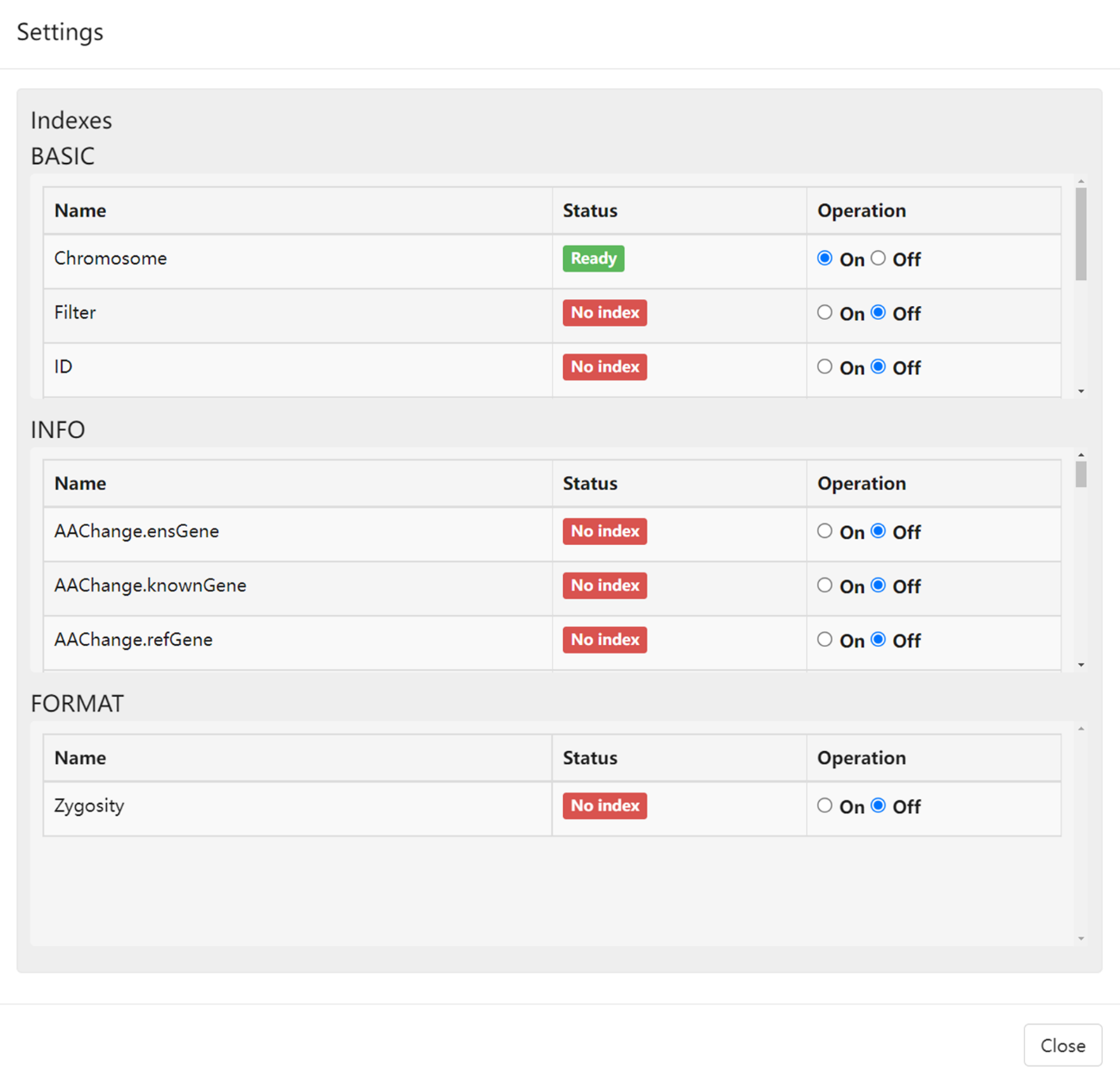


**Figure S12.** The web interface for Setting up the display results for genotypic diagnosis of diseaseGPS. Users can configure the indexing operation for each column.

**
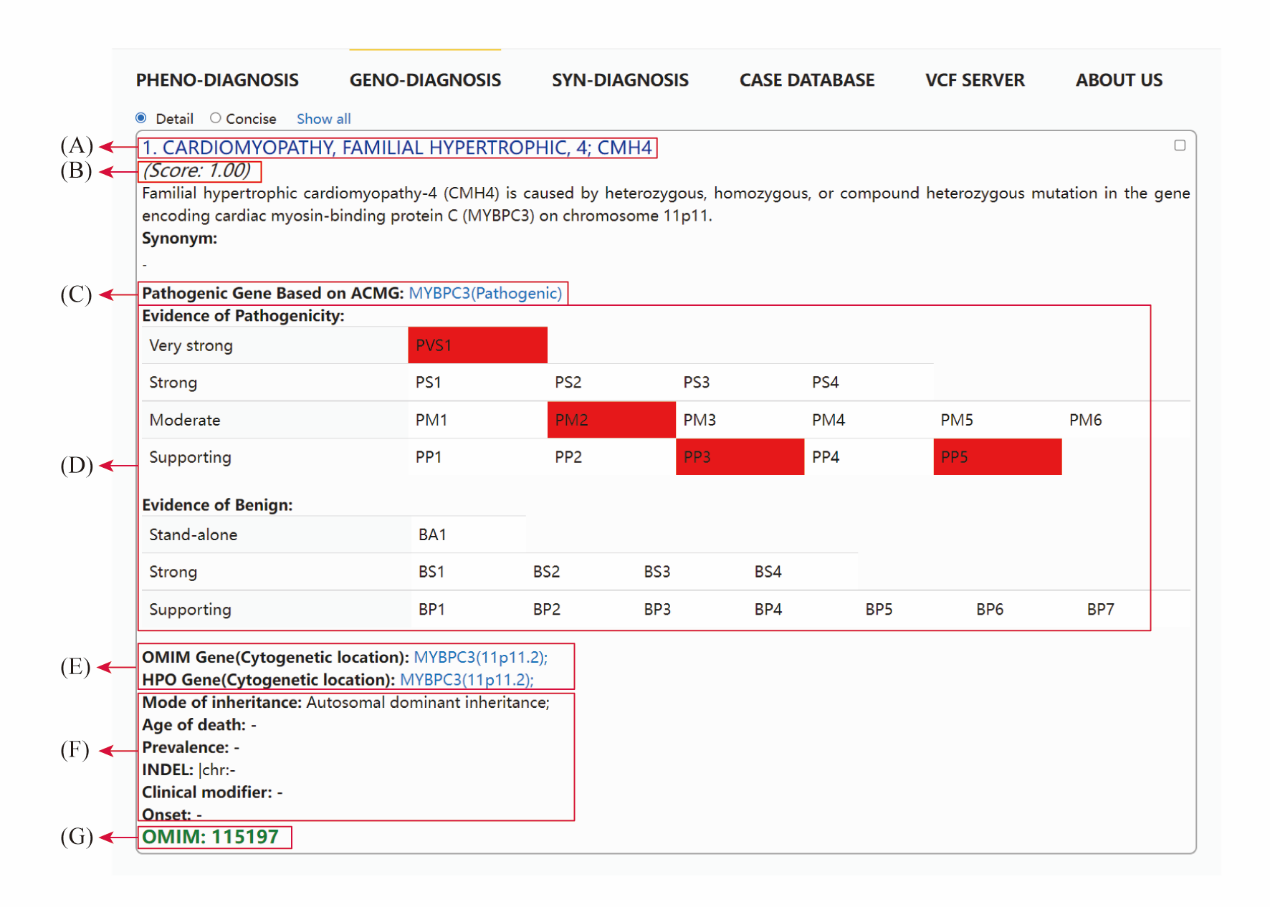
**

**Figure S13.** An example of genotypic diagnosis for diseaseGPS. **(A)** The name of a genetic disorder and its rank among all genetic disorders. Users can click on this item to obtain more detailed information about the genetic disorder. **(B)** The diseaseGPS score of the genetic disorder. **(C)** The pathogenic gene based on ACMG. Users can click on this item to obtain all variant information on the gene, which is equivalent to filtering specific genes in Figure S9. **(D)** The evidence of pathogenicity and benign. All evidences were divided into 28 categories according to ACMG-AMP guidelines. A box with a red background indicates that the presence of evidence. When users hover over the evidence with their mouse, detailed judging criteria will be displayed. **(E)** The causative gene associated with the genetic disorder. Users can click on this item to obtain more detailed information about the causative gene, as shown in Figure S6. **(F)** More information about the genetic disorder, including mode of inheritance, age of death, prevalence, indel clinical modifier and onset. **(G)** The ID of the genetic disorder in the OMIM database. Users can click on this item to access a hyperlink to the OMIM database for more information.

**
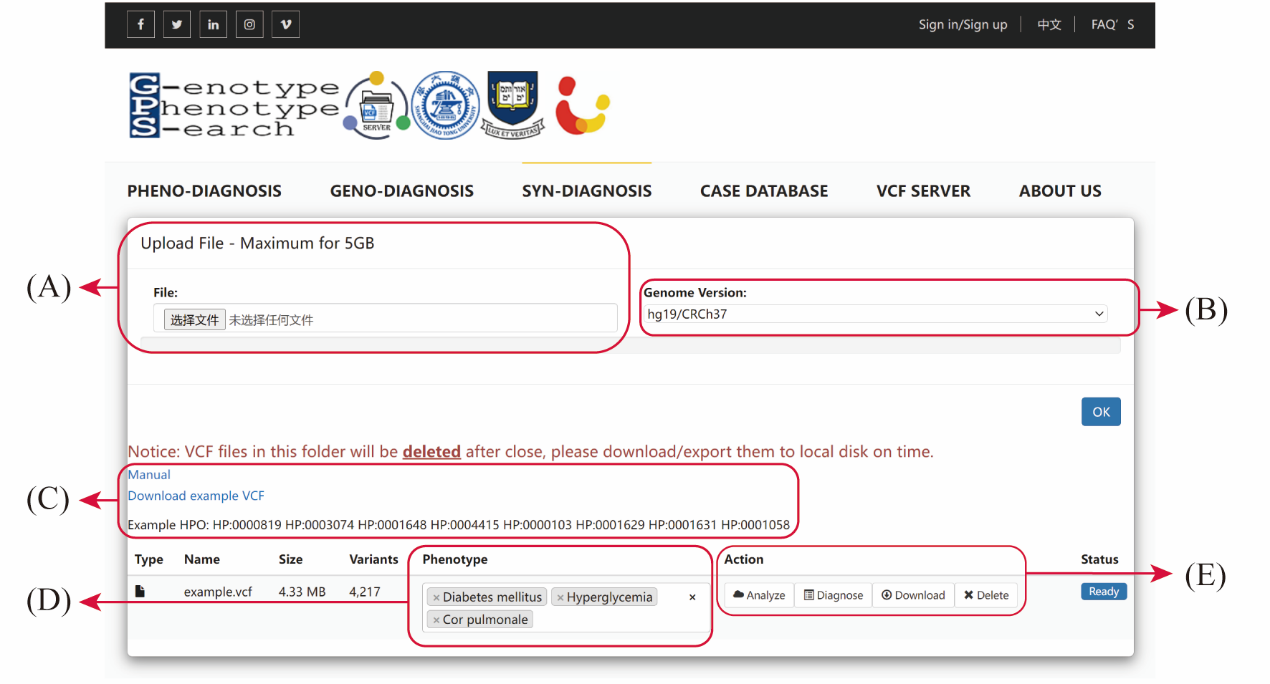
**

**Figure S14.** The web interface for synthesize diagnosis of diseaseGPS. **(A)** The box for uploading VCF files. diseaseGPS accepts VCF files or vcf.gz files up to 5GB in size. **(B)** The genome version selection box. Users can choose hg19/CRCh37 or hg38/CRCh38 according to the annotated genome of VCF file. **(C)** The example VCF file, HPO terms and user manual. Users can click to download the example VCF file and use the VCF file and HPO terms according to the manual. **(D)** The phenotype input box. Users can search by manually entering phenotypes into the phenotype input box. **(E)** The actions that users can select. Users can click the "Analyze" box to obtain the annotation analysis result of the VCF file, as shown in Figure S9. Users can click the "Diagnose" box to obtain the disease diagnosis prediction result of the VCF file and HPO terms, as shown in Figure S15.


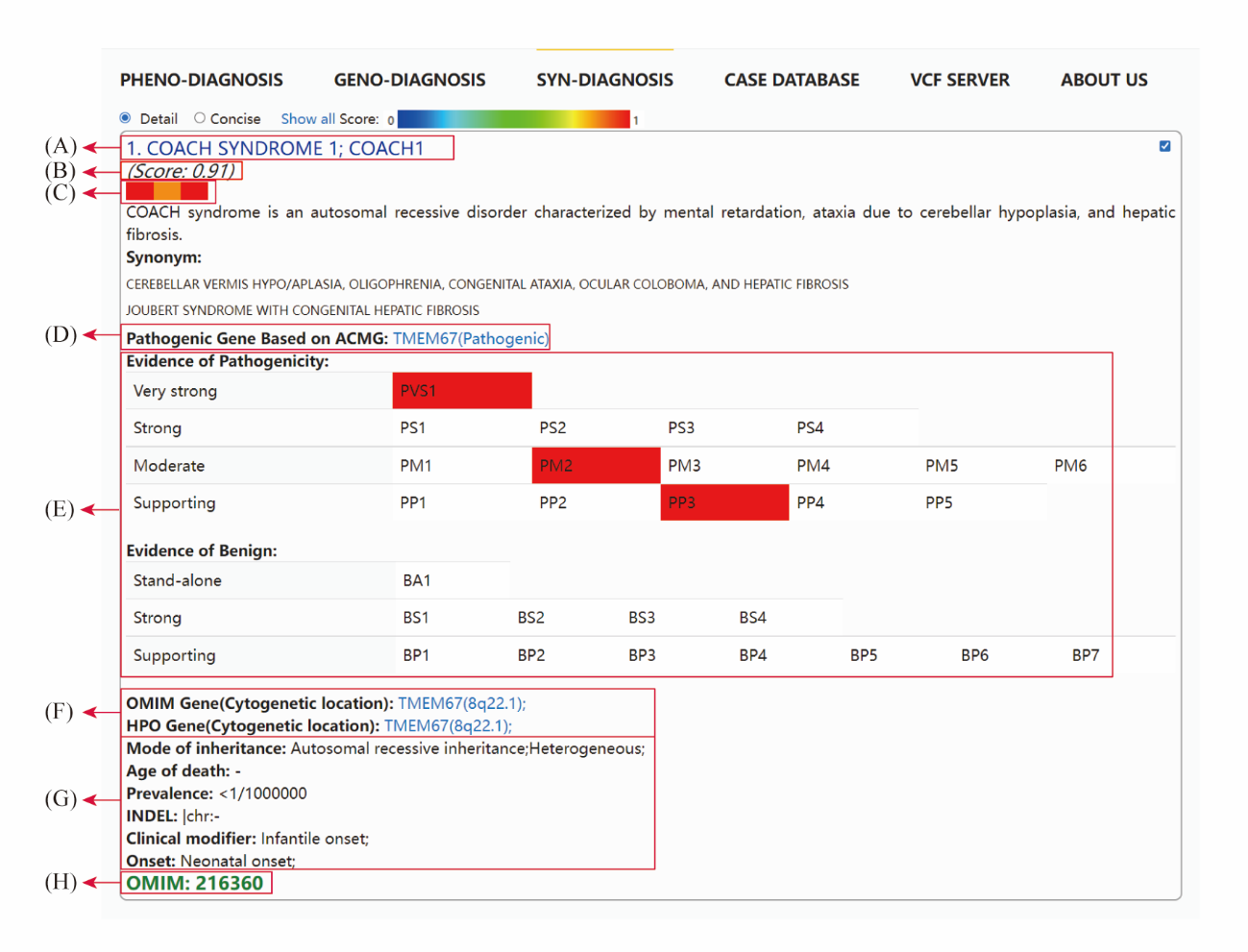


**Figure S15.** An example of synthesize diagnosis for diseaseGPS. **(A)** The name of a genetic disorder and its rank among all genetic disorders. Users can click on this item to obtain more detailed information about the genetic disorder, as shown in Figure S4. **(B)** The synthesize diseaseGPS score of the genetic disorder. **(C)** The correlation between the inputted phenotype and the genetic disorder. As the color changes from blue to red, its correlation increases gradually. Users can click on one of the color blocks to view the phenotype relation diagram for that phenotype in the entire phenotype tree, as shown in Figure S5. **(D)** The pathogenic gene based on ACMG. Users can click on this item to obtain all variant information on the gene, which is equivalent to filtering specific genes in Figure S9. **(E)** The evidence of pathogenicity and benign. All evidences were divided into 28 categories according to ACMG-AMP guidelines. A box with a red background indicates that the presence of evidence. When users hover over the evidence with their mouse, detailed judging criteria will be displayed. **(F)** The causative gene associated with the genetic disorder. Users can click on this item to obtain more detailed information about the causative gene, as shown in Figure S6. **(G)** More information about the genetic disorder, including mode of inheritance, age of death, prevalence, indel clinical modifier and onset. **(H)** The ID of the genetic disorder in the OMIM database. Users can click on this item to access a hyperlink to the OMIM database for more information.


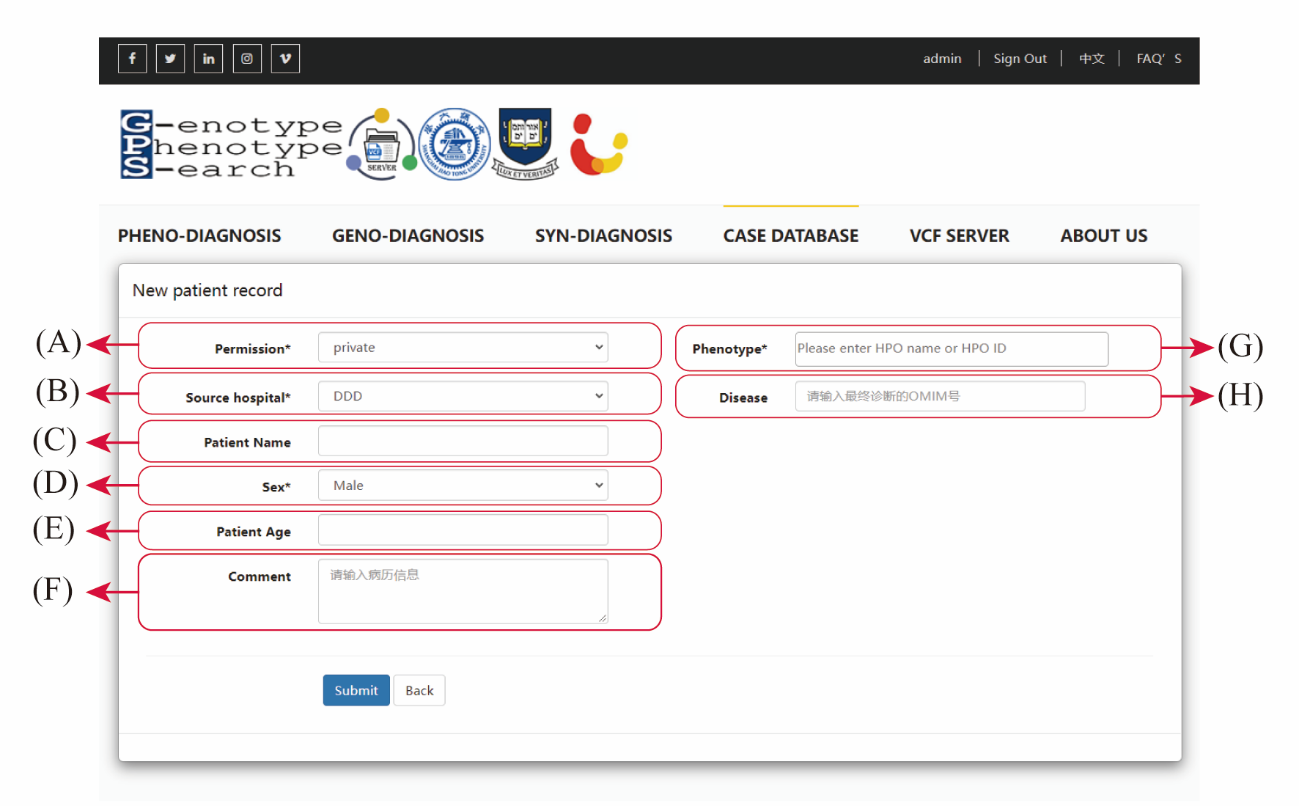


**Figure S16.** Add a new patient record to the case database. It should be noted that users must log in to their account to create their own case database. **(A)** The permission box. Users can choose private or public. **(B)** The source hospital box. **(C)** The patient name box. **(D)** The sex box. Users can choose male, female or other. **(E)** The patient age box. **(F)** The comment box. Users can enter patient medical record information. **(G)** The phenotype box. Users can search by manually entering phenotypes in the phenotype input box. **(H)** The disease box. Users can enter the OMIM number of the patient's final diagnosis of the genetic disorder.


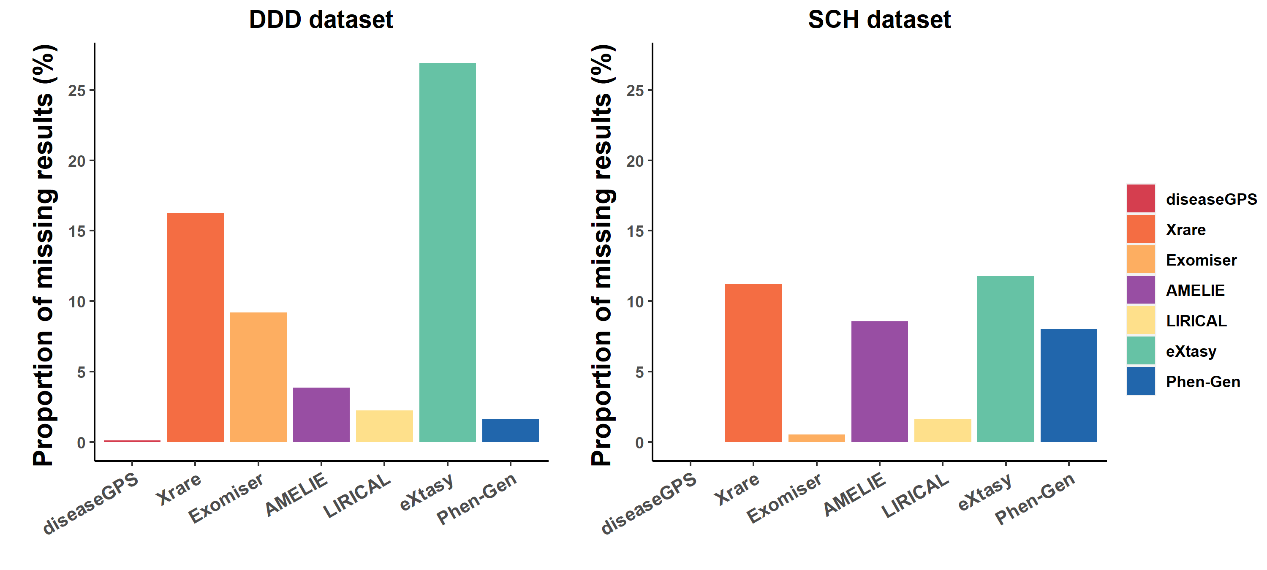


**Figure S17.** Proportion of missing results for each algorithm among all cases in the DDD dataset and SCH dataset. The abscissa represents the names of the various tools. The ordinate represents the proportion of cases, among all cases, where pathogenic variants are filtered out after pre-screening and resulted in missing results.


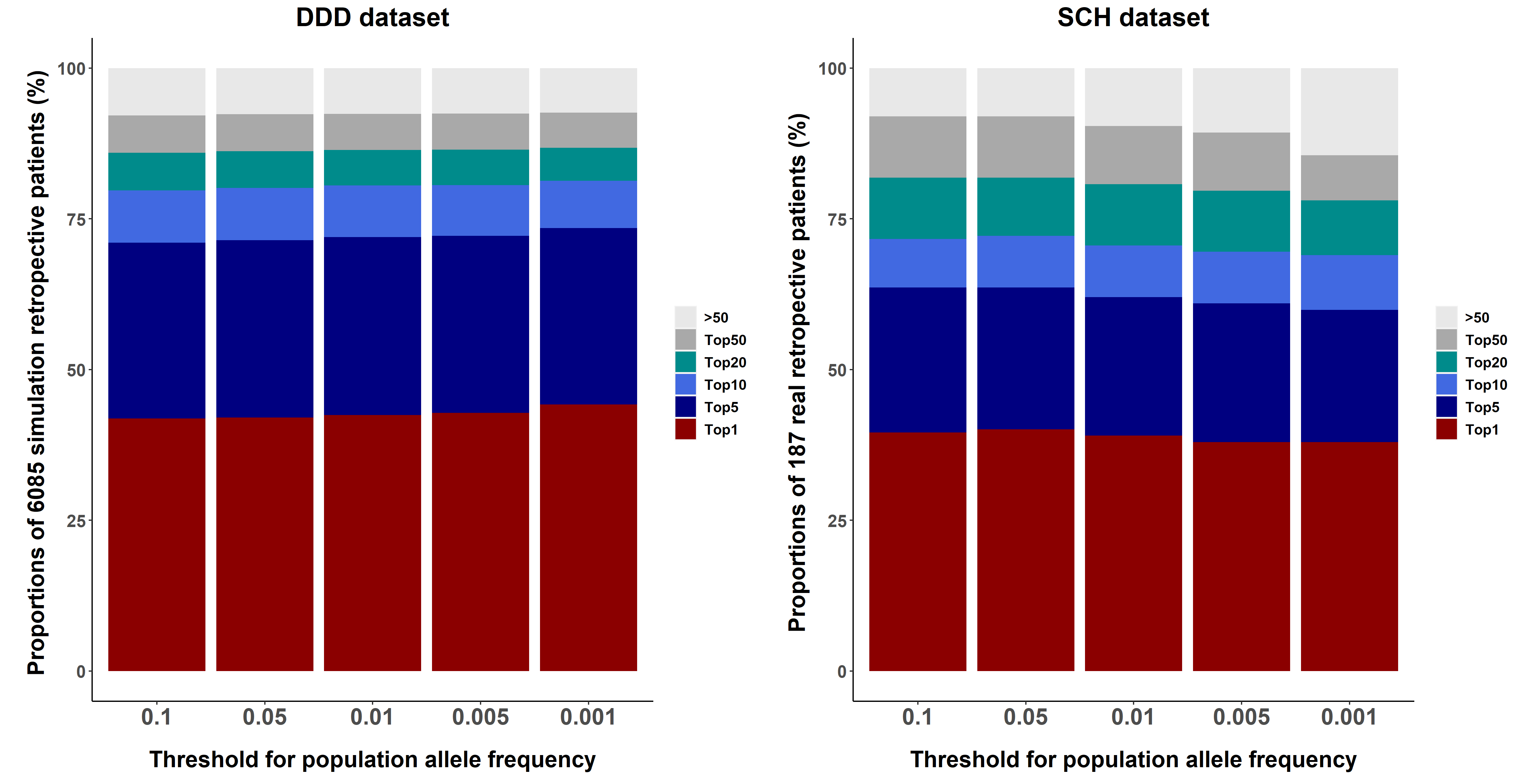


**Figure S18.** Comparison of results of diseaseGPS based on different thresholds for population allele frequency. The abscissa represents the thresholds for population allele frequency and the ordinate represents the proportion of cases with a causal outcome among TOP-1, TOP-5, TOP-10, TOP-20, TOP-50, and >50, respectively.


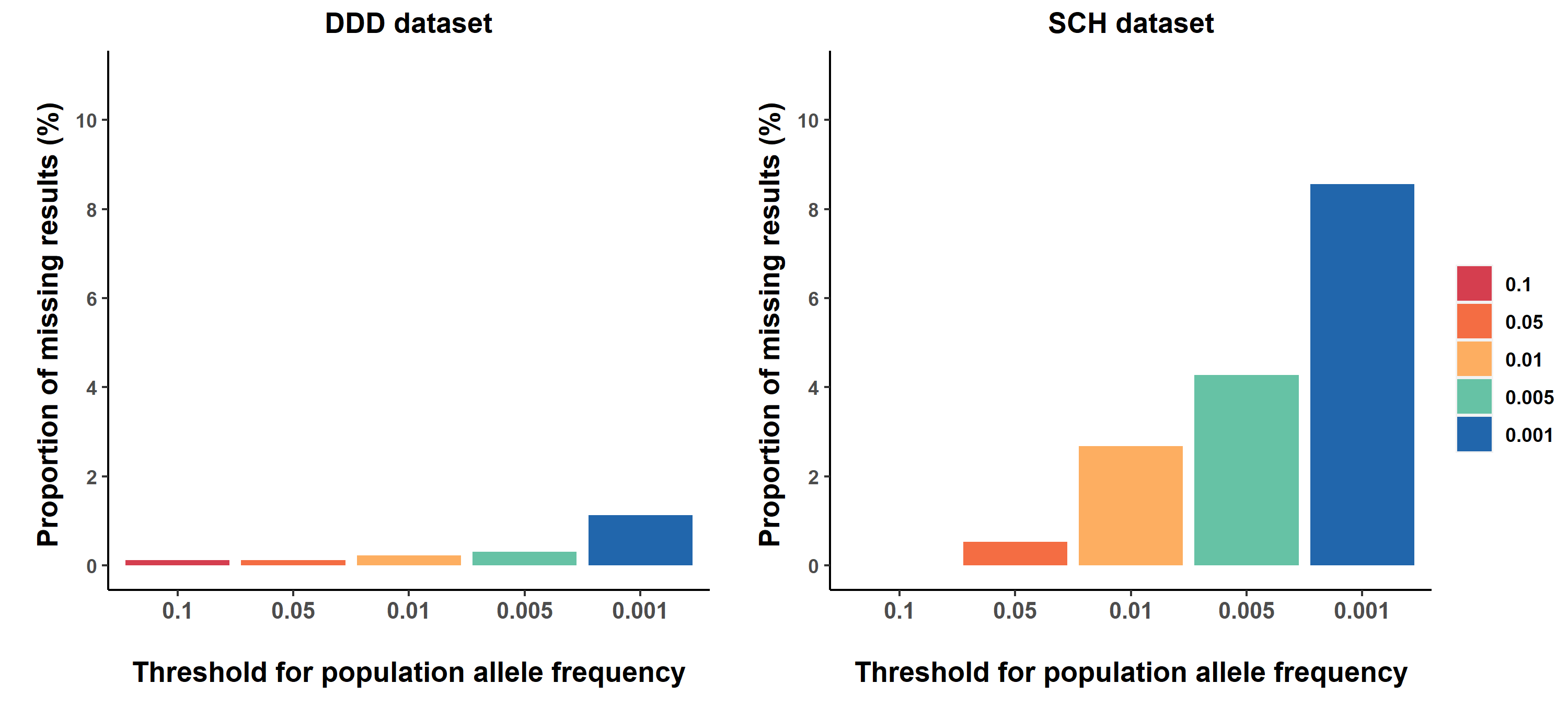


**Figure S19.** Proportion of missing results for diseaseGPS among all cases in the DDD dataset and SCH dataset based on different thresholds for population allele frequency. The abscissa represents the thresholds for population allele frequency. The ordinate represents the proportion of cases, among all cases, where pathogenic variants are filtered out after pre-screening and resulted in missing results.


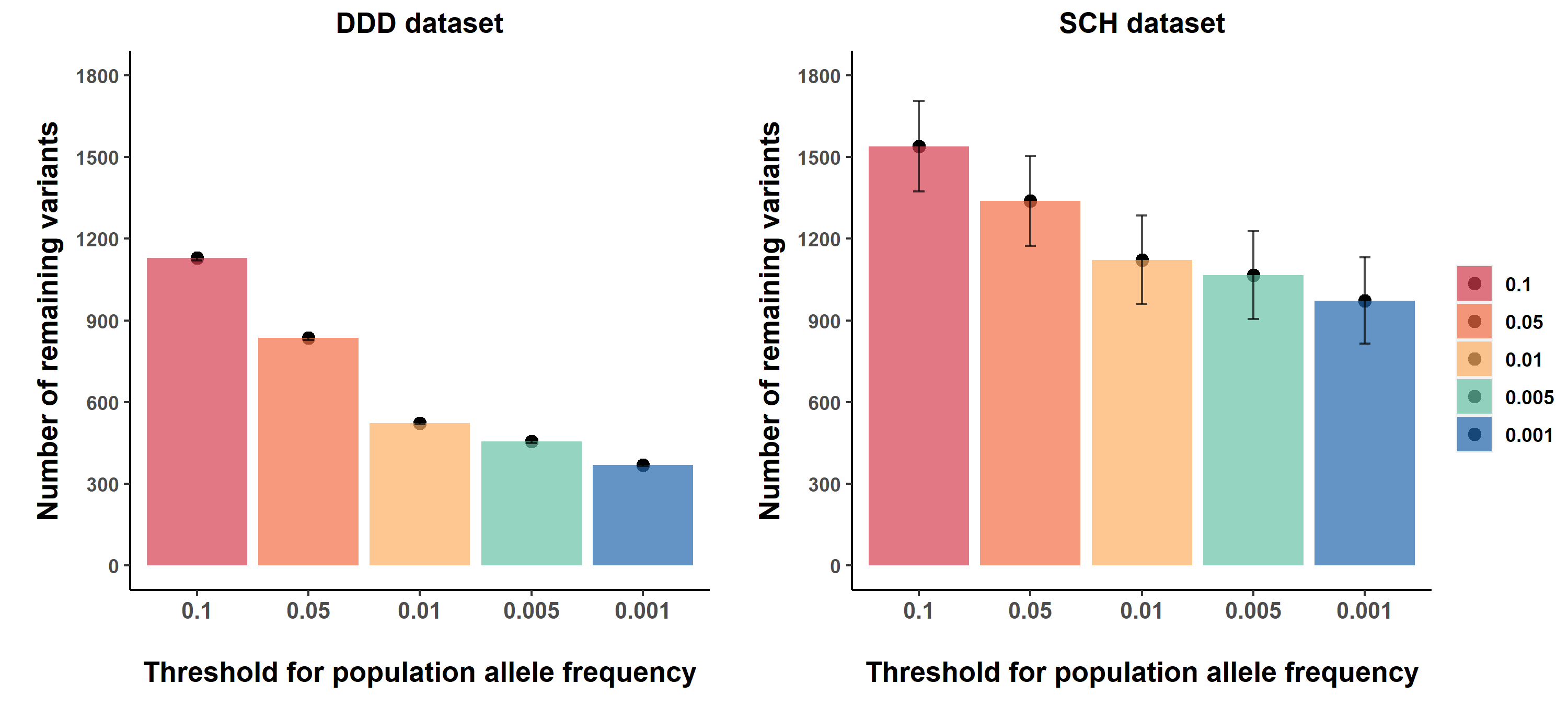


**Figure S20.** Number of remaining variants after pre-screening based on different thresholds for population allele frequency. The abscissa represents the thresholds for population allele frequency. The ordinate represents the remaining number of variants after pre-screening. Due to the use of background genomes from 157 healthy individuals in the DDD dataset, the results presented here were tested using VCF data from these 157 individuals rather than the simulated 6085 cases. The error bars in the graph represent the 95% confidence intervals of the mean.

**
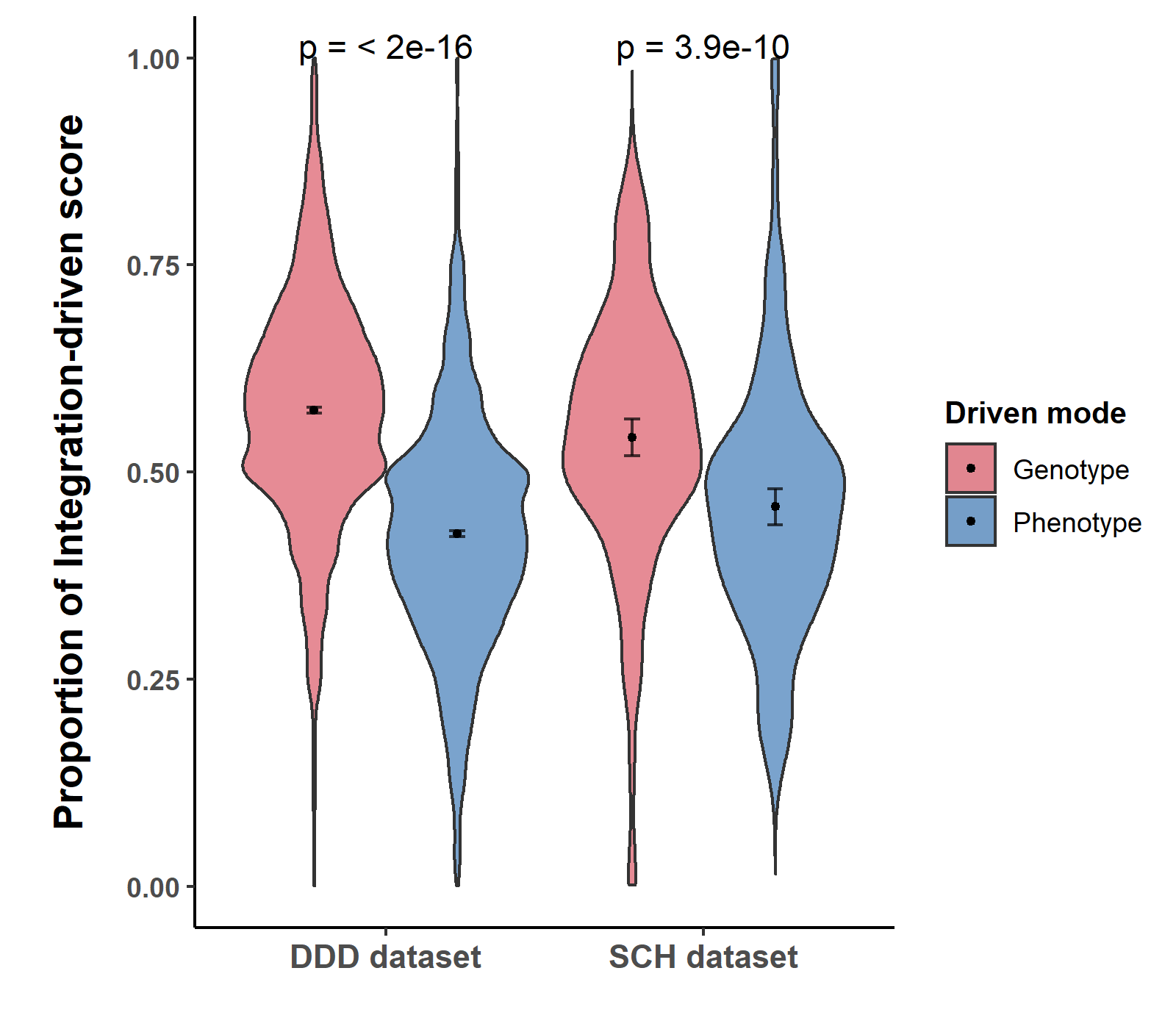
**

**Figure S21**. Proportion of phenotype-driven and genotype-driven scores relative to the integration-driven score. The abscissa represents the dataset in which the data is located. The ordinate represents the proportion of scores relative to the integration-driven score. The legend represents phenotype-driven group and genotype-driven group. The error bars represent the 95% confidence intervals of the mean. The p-value indicates the result of the Wilcoxon signed-rank test comparing the phenotype-driven group and the genotype-driven group.

**
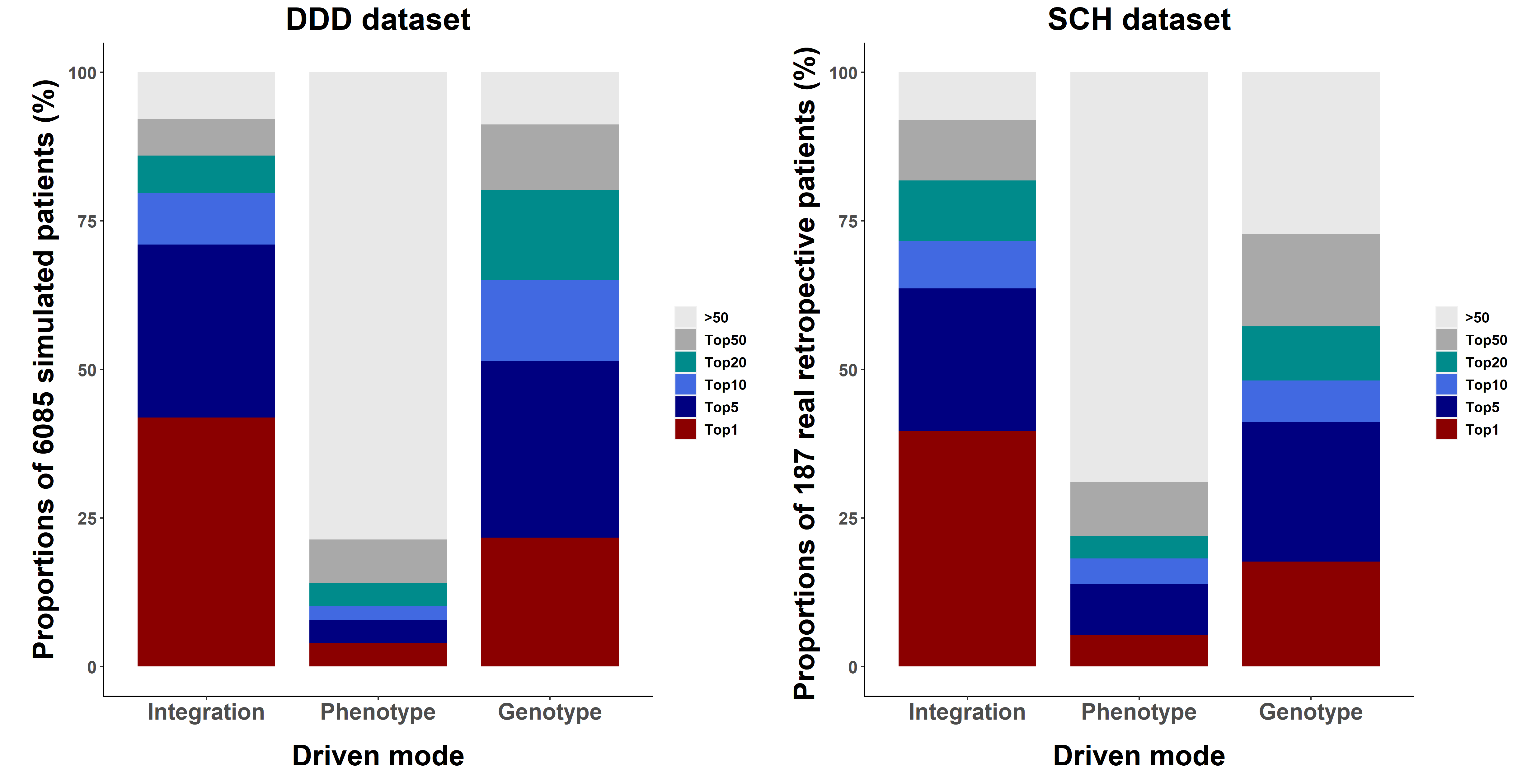
**

**Figure S22.** Proportion of ranking results under different driving modes. The abscissa represents the driven mode. The ordinate represents the proportion of cases with a causal outcome among TOP-1, TOP-5, TOP-10, TOP-20, TOP-50, and >50, respectively.

**Supplementary Discussion**

diseaseGPS is an auxiliary diagnosis system for genetic disorders that integrates phenotypic data and genotype data. Due to the variability of patient data, we provide three different diagnostic methods, including phenotype-driven diagnosis, genotype-driven diagnosis, and phenotype- and genotype-driven diagnosis. For genotype-driven diagnosis, we utilized the evidence framework based on the ACMG-AMP guidelines(Richards, et al., 2015). The phenotype-driven diagnostic approach has been described in detail in the main text.

To compare diseaseGPS with other software more clearly, we plotted the rankings of causative genes for TOP-1, TOP-5, TOP-10 and TOP-20 by diseaseGPS and two other tools, Xrare (Li, et al., 2019) and Exomiser (Robinson, et al., 2014), as shown in Figure S1. The Venn diagram for TOP-1 shows that diseaseGPS and Xrare share many of the same sorting results, but there are also many different, possibly due to commonalities and differences between the two algorithms. In addition to pathogenicity information of the variant site and semantic similarity scores of the phenotype, Xrare also includes a lot of gene-related features, and its algorithm effect is significantly different from the training data. In comparison, diseaseGPS and Exomiser share more of same ranking results than different ones, since both use gene and phenotype scores and have certain similarities. The Exomiser tool uses the phenotype score that incorporates the phenotype of the model organism mouse, which adds a certain degree of difference. As the sorting requirements relax from TOP-1 to TOP-5, TOP-10 and TOP-20, the number of same sorting results also increases. This situation is consistent with the actual clinical diagnosis of genetic disorders. However, doctors often only consider the results that rank high, and the reference value of the results that rank low will significantly decrease.

**Comparison of missing result proportions among different tools**

The missing rate of results is an important indicator for major causal gene sorting algorithms. For an individual patient, if the causative gene or disease diagnosis is missing in the final recommendation system, it can lead to a serious diagnostic error. The missing results of various causal gene sorting algorithms can be attributed to two main factors: pre-screening thresholds that are set too high, leading to the loss of some results and insufficient information in the databases, which results in the loss of some additional results. The proportion of missing results for each algorithm is shown in Figure S17. diseaseGPS has a significantly lower proportion of missing results in both the DDD dataset and SCH dataset compared to other algorithms. The prediction performance of eXtasy (Sifrim, et al., 2013) and Phen-Gen (Javed, et al., 2014) in the main text is poor, and they also have a high proportion of missing results, possibly due to using an old database. Xrare (Li, et al., 2019), Exomiser (Robinson, et al., 2014), AMELIE (Birgmeier, et al., 2020) and LIRICAL (Robinson, et al., 2020) have good prediction performance, but their proportion of missing results is not low, which is related to the high threshold they set. The pre-screening threshold in diseaseGPS is conservative, leading to higher accuracy of prediction results and lower proportion of missing results.

**Exploration of pre-screening threshold settings**

To investigate the relationship between the threshold settings of pre-screening and the final ranking results, we conducted additional experiments, as shown in Figures S18-S20. Figure S18 illustrates that in the DDD dataset, decreasing the population allele frequency threshold from 0.1 to 0.001 resulted in a slight improvement in the final ranking performance. Conversely, a decrease in the population allele frequency threshold from 0.1 to 0.001 led to a slight decline in the final ranking performance. The Friedman test yielded a p-value < 2.2e-16 for the DDD dataset and a p-value < 2.2e-16 for the SCH dataset, indicating a significant association between the population allele frequency threshold and the ranking results. This phenomenon can be attributed to the presence of distinct key pathogenic variants that vary between the two datasets. Figure S19 demonstrates that as the population allele frequency threshold decreased from 0.1 to 0.001, the proportion of missing results gradually increased in both the DDD and SCH datasets. However, the proportion of missing results in the DDD dataset was much lower than that in the SCH dataset. When key pathogenic variants are filtered out, the final ranking results for those cases become unavailable. The SCH dataset exhibited a significantly higher proportion of filtered pathogenic variants than the DDD dataset, resulting in improved performance for the DDD dataset when the filtering threshold was lowered, while the SCH dataset experienced a performance decline.

As depicted in Figure S20, decreasing the population allele frequency threshold from 0.1 to 0.001 resulted in a gradual reduction in the number of remaining variants after pre-screening. Prior to the pre-screening step, the VCF file of the DDD dataset, comprising 157 healthy individuals, had an average of 7213.9236 variants, with a 95% confidence interval of [7174.1643, 7253.682926]. The VCF file of the SCH dataset consisting of 187 cases, had an average of 45273.3262 variants, with a 95% confidence interval of [41934.6705, 48611.9819]. Following pre-screening, the SCH dataset experienced a greater reduction in the number of variants compared to the DDD dataset. This decrease in variant count can alleviate computational burden and complexity in subsequent analysis and processing stages. The reduction in variants, along with the proportion of filtered key variants, collectively impact the final ranking results. The purpose of implementing the pre-screening step is to exclude irrelevant common variants, while minimizing the risk of filtering out potential pathogenic variants. Hence, a relatively conservative population allele frequency threshold of 0.1 was chosen instead of a lower threshold. diseaseGPS is an openly accessible and free platform for genetic disorder diagnosis. Filtering out patients' pathogenic variants directly would cause significant confusion and distress among users. However, if users have specific requirements, they can utilize a localized version of diseaseGPS to set a lower population allele frequency threshold.

**Exploration of weights for phenotype-driven and genotype-driven approaches**

diseaseGPS comprises two components: phenotype-driven and genotype-driven. The weighting of these components in the integrated score enhances users' comprehension of the diseaseGPS architecture. Figure S21 illustrates the proportions of phenotype-driven and genotype-driven scores within the overall composite scores for each case. It is be observed that the proportions of phenotype-driven and genotype-driven scores are similar in both the DDD dataset and the SCH dataset. In the DDD dataset, the average proportion of phenotype-driven scores is 0.4256821, with a 95% confidence interval of [0.4222012, 0.4291630]. Conversely, the average proportion of genotype-driven scores is 0.5743179, with a 95% confidence interval of [0.5777988, 0.570837]. Within the SCH dataset, the average proportion of phenotype-driven scores is 0.4580004, with a 95% confidence interval of [0.4360847, 0.4799161]. Similarly, the average proportion of genotype-driven scores is 0.5743179, with a 95% confidence interval of [0.5200839, 0.5639153]. The proportion of genotype-driven scores is significantly higher than that of phenotype-driven scores, as indicated by the p-value. However, the difference between the proportions of phenotype-driven and genotype-driven scores is not substantial. Both phenotype data and genotype data play crucial roles in the final ranking results, with genotype data having slightly greater importance. This observation aligns with the existing genetic disorder diagnostic process, where the confirmation of a genetic disorder necessitates the presence of associated pathogenic variants. If a patient exhibits a strongly correlated phenotype for a specific genetic disorder but lacks corresponding pathogenic variants in the associated genes, there is a higher likelihood of misdiagnosis. Therefore, it is important to consider other potentially overlapping genetic disorders and their associated pathogenic variants.

diseaseGPS supports an auxiliary diagnostic mode that integrates phenotypic and genotypic drivers. It also supports a diagnostic mode driven by a single type of data. However, we highly recommend users to utilize the integration-driven mode for auxiliary diagnosis. As shown in Figure S22, the integration-driven mode outperforms the single-data driven mode, with a Friedman test yielding a p-value less than 2.2e-16 in both the DDD dataset and SCH dataset. However, it is important to note that pathogenic variants do not always manifest in patients and are influenced by various factors. Therefore, while pathogenic variants possess high pathogenicity scores, integrating them with phenotypic information in a diagnostic context can provide a more precise determination of the genetic disorder affecting the patient. Genetic disorders often exhibit numerous phenotypic similarities, which is why relying solely on phenotype-driven approaches yields suboptimal results. This aspect has been discussed in the supplementary methods section, specifically regarding phenotypic similarity. Phenotypic data aid in identifying the phenotypic manifestation of pathogenic variants, while variant data help eliminate confounding phenotypes. The combination of phenotypic and genotypic data creates a synergistic effect, resulting in outcomes that surpass the individual impacts of each data type.

To further enhance the prediction accuracy of genetic disorders and to help doctors and patients better understand the basis of the algorithm's judgment, an explicit display of the pathogenic mechanism from variant to phenotype could be the focus of future work.

**Supplementary References**

Birgmeier, J., et al. AMELIE speeds Mendelian diagnosis by matching patient phenotype and genotype to primary literature. Science Translational Medicine 2020;12(544).

Blanchard, E., Harzallah, M. and Kuntz, P. A generic framework for comparing semantic similarities on a subsumption hierarchy. In, 18th European Conference on Artificial Intelligence. Univ Patras, Patras, GREECE; 2008. p. 20-24.

Fitzgerald, T.W., et al. Large-scale discovery of novel genetic causes of developmental disorders. Nature 2015;519(7542):223-+.

Hamosh, A., et al. Online Mendelian Inheritance in Man (OMIM), a knowledgebase of human genes and genetic disorders. Nucleic Acids Research 2005;33:D514-D517.

Javed, A., Agrawal, S. and Ng, P.C. Phen-Gen: combining phenotype and genotype to analyze rare disorders. Nature Methods 2014;11(9):935-937.

Lappalainen, I., et al. The European Genome-phenome Archive of human data consented for biomedical research. Nature Genetics 2015;47(7):692-695.

Li, Q., et al. Xrare: a machine learning method jointly modeling phenotypes and genetic evidence for rare disease diagnosis. Genetics in Medicine 2019;21(9):2126-2134.

Richards, S., et al. Standards and guidelines for the interpretation of sequence variants: a joint consensus recommendation of the American College of Medical Genetics and Genomics and the Association for Molecular Pathology. Genet Med 2015;17(5):405-424.

Robinson, P.N., et al. The Human Phenotype Ontology: A Tool for Annotating and Analyzing Human Hereditary Disease. American Journal of Human Genetics 2008;83(5):610-615.

Robinson, P.N., et al. Improved exome prioritization of disease genes through cross-species phenotype comparison. Genome Research 2014;24(2):340-348.

Robinson, P.N., et al. Interpretable Clinical Genomics with a Likelihood Ratio Paradigm. American Journal of Human Genetics 2020;107(3):403-417.

Sifrim, A., et al. eXtasy: variant prioritization by genomic data fusion. Nature Methods 2013;10(11):1083-1084.

Tavtigian, S.V., et al. Modeling the ACMG/AMP variant classification guidelines as a Bayesian classification framework. Genetics in Medicine 2018;20(9):1054-1060.

Yuan, X., et al. Evaluation of phenotype-driven gene prioritization methods for Mendelian diseases. Briefings in Bioinformatics 2022;23(2).
